# Thermoresponsive Nanoparticles with Cyclic-Polymer-Grafted Shells Are More Stable than with Linear-Polymer-Grafted Shells: Effect of Polymer Topology, Molecular Weight, and Core Size

**DOI:** 10.1021/acs.jpcb.1c00142

**Published:** 2021-06-22

**Authors:** Max Willinger, Erik Reimhult

**Affiliations:** Institute for Biologically Inspired Materials, Department of Nanobiotechnology, University of Natural Resources and Life Sciences Vienna, Muthgasse 11, 1190 Vienna, Austria

## Abstract

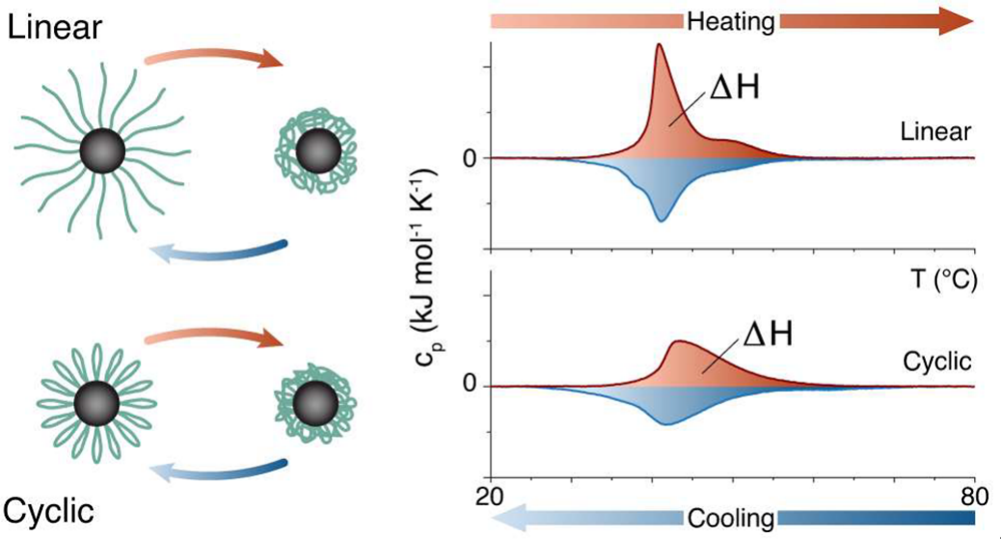

Polymer brush-grafted superparamagnetic iron oxide nanoparticles can change their aggregation state in response to temperature and are potential smart materials for many applications. Recently, the shell morphology imposed by grafting to a nanoparticle core was shown to strongly influence the thermoresponsiveness through a coupling of intrashell solubility transitions and nanoparticle aggregation. We investigate how a change from linear to cyclic polymer topology affects the thermoresponsiveness of poly(2-isopropyl-2-oxazoline) brush-grafted superparamagnetic iron oxide nanoparticles. Linear and cyclic polymers with three different molecular weights (7, 18, and 24.5 kg mol^–1^) on two different core sizes (3.7 and 9.2 nm) and as free polymer were investigated. We observed the critical flocculation temperature (CFT) during temperature cycling dynamic light scattering experiments, the critical solution temperature (CST), and the transition enthalpy per monomer during differential scanning calorimetry measurements. When all conditions are identical, cyclic polymers increase the colloidal stability and the critical flocculation temperature compared to their linear counterparts. Furthermore, the cyclic polymer shows only one uniform transition, while we observe multiple transitions for the linear polymer shells. We link the single transition and higher colloidal stability to the absence in cyclic PiPrOx shells of a dilute outer part where the particle shells can interdigitate.

## 1 Introduction

Thermoresponsive colloidal smart materials received tremendous attention in recent years. They are investigated for use not only in medical applications,^1^ such as drug delivery,^2^ hyperthermia,^3^ or as contrast agents,^4^ but also for catalysis,^5^ sensing,^6^ or separation.^7^ It is crucial to know the nanoparticle colloidal system’s phase diagram under realistic conditions to optimize such applications. Many parameters influence the critical solution temperature (CST) of the particles’ polymer coating, which controls the thermoresponsive behavior in all such applications. These parameters include the concentration,^8^ the end group,^9^ the monomer composition,^10^ the ionic strength and type of the aqueous surrounding,^8^ and, as recently demonstrated, the local monomer concentration determined by the polymer shell morphology,^8,11^ which also is influenced by the nanoparticle curvature.^12,13^

Colloidal nanoparticles investigated for thermoresponsive applications include hydrogels,^14^ micelles,^15,16^ vesicles,^17,18^ as well as inorganic-polymer hybrid particles.^19−21^ Inorganic-polymer hybrid particles are particularly interesting. They can combine the unique optical, magnetic, and electric properties of inorganic nanomaterials with the thermally induced phase transitions of hydrated polymers.^22,23^ The most well-defined inorganic-polymer hybrid nanoparticle is the core–shell nanoparticle, comprising an inorganic core with a grafted thermoresponsive polymer shell.^24−27^

So far, the investigations of the thermoresponsiveness of core–shell nanoparticles have been limited to particles with a hydrogel-like coating or, more commonly, a polymer brush of linear polymers. Recent studies showed that this seemingly simple system could demonstrate a complex phase diagram for the polymer solvation transitions within the brush, resulting from the highly curved geometry imposed on the polymer brush.^28^ The change in segment density and thereby free volume per monomer radially from the core surface shifts the thermal transitions consistent with the concentration dependence of the solvation transition.^13,29^ More surprisingly, it was shown that instead of a gradual broadening of the overall transition of the brush, different density regimes within the brush lead to distinguishable transitions. However, the colloidal aggregation resulting from a loss of shell hydration and osmotic repulsion, i.e., the critical flocculation temperature (CFT), can be ascribed to one of the brush transitions. It is not clear from current literature which section of the brush triggers the flocculation. Collapses of both the inner and the outermost parts of the brush have been proposed to correlate with nanoparticle aggregation.^8,13^ This might be explained by that nanoparticle aggregation is predominantly driven by the core–core van der Waals attraction; even above the critical solution temperature, the investigated polymer brushes remain mostly hydrated and could act osmotically repulsive. These findings imply that alterations to the brush density profile and changes to the stabilizing polymer brushes’ ability to interpenetrate could strongly influence the thermoresponsive behavior of core–shell nanoparticle dispersions.

Recently, polymer brushes with a cyclic topology were introduced for biointerface applications.^30,31^ The approach got a boost by advances in the synthesis of cyclic polymers suitable for surface modification in biological fluids.^32^ Cyclic polymers improve the brush density and reduce the interpenetration of polymers on opposing polymer-grafted surfaces.^33^ The motivation for this approach harks back to theoretical studies suggesting a significantly lower probability of cyclic polymer brushes penetrating each other and a higher polymer segment density close to the surface than for linear polymer brushes.^30^ Additionally, experimental findings and theory suggest a lower entropic penalty to surface adsorption for cyclic than linear polymers, leading to denser adsorbed polymer films^34−36^ and higher grafting densities achieved.^37^ These advantageous properties offset the decreased peak repulsive potential of cyclic polymer compared to linear polymer brushes at the same molecular and grafting density. This trade-off can provide significant advantages when the repulsive potential of cyclic polymer brushes still is sufficient to prevent aggregation with other colloids. Polymers, e.g., biopolymer such as proteins, can penetrate dynamic defects in less dense linear brush shells and allow strong adsorption within the shell. Thus, it was recently shown that cyclic polymer brush shells on small iron oxide nanoparticles provide qualitatively and quantitatively improved colloidal stability and protection against the adsorption of human serum albumin compared to equivalent linear polymer brush shells.^32,37,38^

Cyclic polymers have previously been investigated for their thermal stability as micelles. Tezuka and co-workers described higher thermal stability of cyclic block copolymers compared to chemically identical linear ABA triblock copolymers.^34^ This observation was tentatively explained as a lower entropic penalty for cyclic polymers to participate in micelle formation and a reduced ability to do bridging between micellar cores without two free ends.

In this work, we investigate the influence of cyclic polymer topology on the CST of the stabilizing shells and the CFT of nanoparticle dispersions of superparamagnetic iron oxide cores grafted with dense poly(2-isopropyl-2-oxazoline) (PiPrOx) brush shells. Using our established approach from investigating the effect of polymer shell morphology on thermal responsiveness,^28,32^ we compare results from differential scanning calorimetry (DSC) and dynamic light scattering (DLS) to determine the interplay between internal polymer shell properties and overall dispersion properties as a function of temperature. We refer to the measurement of the solvation transitions of the polymer brushes using DSC as corresponding to the CST of the polymer. Measurements of particle aggregation using DLS are referred to as defining the CFT of the core–shell nanoparticle dispersion.

These investigations were performed for 3.7 and 9.2 nm iron oxide cores and three different molecular weights (7, 18, and 24.5 kg mol^–1^) of linear and cyclic PiPrOx, respectively, and compared to the free polymers. The grafting densities of the polymer brushes bound to each particle were carefully controlled to be similar, using methods developed previously for ligand replacement,^39^ irreversible grafting,^40^ and purification.^10^ We did this to ensure that the effects of polymer molecular weight (shell thickness) and topology could be distinguished from grafting density.

## 2 Materials and Methods

### 2.1 Materials

Dopamine hydrochloride, sodium nitrite, sulfuric acid (98%), sodium azide, 2-bromoethylamine hydrobromide, isobutyronitrile, ethanolamine, zinc acetate dihydrate, anhydrous *N,N*-dimethylacetamide (DMA), copper bromine (CuBr), sodium ascorbate, succinic anhydride, triethylamine (TEA), 4-(dimethylamino)pyridine (DMAP), *N,N*-diisopropylethylamine (DIPEA), and anhydrous *N,N*-dimethylformamide (DMF) were purchased from Merck KGaA (Darmstadt, Germany) and were used without any purifications. Methyl-*p*-tosylate (MeTs) and propargyl *p*-toluenesulfonate (PrTs) were purchased from Merck KGaA (Darmstadt, Germany) and purified by distillation. 1-[(1-(Cyano-2-ethoxy-2-oxoethylidenaminooxy)dimethylaminomorpholino)]uronium hexafluorphosphate (COMU), potassium hydroxide (KOH), ethanol, diethyl ether (Et_2_O), dichloromethane (DCM), hexane, and dry chloroform were purchased from Carl Roth (Karlsruhe, Germany) and were used as received. Dialysis tubes with different molecular weight cutoffs (3.5 kDa regenerated cellulose and 1000 kDa cellulose ester, Spectra/Por Float-A-Lyzer) were purchased from Carl Roth.

### 2.2 Synthesis of Iron Oxide Nanoparticles

Monodisperse iron oxide nanoparticles were synthesized as described previously,^41^ adapted from the method developed by Hyeon et al.^42^ Briefly, iron oxide nanoparticles with average core diameters of 3.7 and 9.2 nm (Figure S1), respectively, were prepared by the thermal decomposition of the precursor iron(0) pentacarbonyl in the presence of oleic acid as the surfactant in dioctyl ether.

### 2.3 Synthesis of 2-Azidoethylamine

2-Bromoethylamine hydrobromide (8.54 g, 41.7 mmol) and 8.46 g (130 mmol) of sodium azide were dissolved in 30 mL of water which was previously deoxygenized in a nitrogen gas atmosphere. The mixture was stirred at 80 °C under nitrogen atmosphere for 16 h. The reaction was quenched with 15.5 M aqueous KOH solution (15 mL). The product was dried over Na_2_SO_4_ after extraction three times with Et_2_O. Yield: 3.12 g (36.2 mmol; 87%). ^1^H NMR (300 MHz; CDCl_3_) δH: 3.36 (t, 2H), 2.88 (t, 2H), 1.42 (s, 1H) (Figure S2).

### 2.4 Synthesis of 6-Nitrodopamine (NDA)

5.0 g (26 mmol) of dopamine hydrochloride and 6.4 g (93 mmol) of sodium nitrite were dissolved in 150 mL of water (deoxygenized in a nitrogen atmosphere). Twenty-five milliliters of 20% sulfuric acid was slowly added after the mixture was cooled to 0 °C. The reaction was warmed to room temperature and stirred for 16 h. The formed dark yellow precipitates were collected by filtration and washed several times with cold (4 °C) water. The product was resuspended in ethanol at 40 °C and precipitated with Et_2_O. Yield: 5.0 g (25 mmol; 95%). ^1^H NMR (300 MHz; DMSO-*d*_6_) δH: 7.45 (s, 1H), 6.78 (s, 1H), 3.04 (m, 4H) (Figure S3).

### 2.5 Synthesis of 2-Isopropyl-2-oxazoline (iPrOx)

The monomer was synthesized using a modified Witte-Seelinger cyclocondensation.^43,44^ Isobutyronitrile (90 mL, 1 mol) was mixed with 72 mL (1.2 mol) of ethanolamine in the presence of 4.4 g (0.02 mol) of zinc acetate dihydrate as the catalyst at 130 °C for 24 h. The product was dissolved in DCM, followed by extraction with water until the pH of the aqueous phase was neutral. It was dried over calcium hydride and distilled in a nitrogen atmosphere to yield an anhydrous product.

### 2.6 Polymerization of 2-Isopropyl-2-oxazoline

The synthesis of poly(2-isopropyl-2-oxazoline) (PiPrOx) was performed in a glovebox under a nitrogen atmosphere. In a flame-dried flask, a 25 (v/v)% solution of 2-isopropyl-2-oxazoline (3 mL, 25.19 mmol, [M] = 2.10 mol L^–1^) in anhydrous *N,N*-dimethylacetamide (DMA) (9 mL) was mixed with the desired amount of the initiator (MeTs: 62 μL (0.41 mmol, [I] = 34.24 mmol L^–1^, [M]:[I] = 61.3), 24 μL (0.16 mmol, [I] = 13.25 mmol L^–1^, [M]:[I] = 158.4), and 17 μL (0.11 mmol, [I] = 9.39 mmol L^–1^, [M]:[I] = 223.6); PrTs: 71 μL (41.0 mmol, [I] = 34.19 mmol L^–1^, [M]:[I] = 61.4), 28 μL (0.16 mmol, [I] = 13.48 mmol L^–1^, [M]:[I] = 155.7), and 20 μL (0.12 mmol, [I] = 9.63 mmol L^–1^, [M]:[I] = 217.9)) and stirred at 100 °C for 24 h. The reaction was quenched with 10 equiv of the desired quencher (0.1 M aqueous NaOH solution for MeTs-initiator, OH-terminated polymer; 2-azidoethylamine for PrTs-initiator, N_3_-terminated polymer). The poly(2-isopropyl-2-oxazoline) was isolated by precipitation with Et_2_O and hexane (ratio 1:1). The MW and PDI were determined by gel permeation chromatography (GPC) against polystyrene standards (Table 1 and Figure S4) for the linear polymer chains.

**Table 1:** Characteristics of Poly(2-isopropyl-2-oxazoline) (PiPrOx)-Grafted Superparamagnetic Iron Oxide Nanoparticles Investigated for Their Thermoresponsive Properties^a^

sample	core size [nm]	topology	mol wt, Mn of linear polymer chains [kg mol^–1^] (DP)	PDI of the linear polymer chains	σ (grafting density) [molecules nm^–2^]
C4MW7-linear	3.7	linear	7 (61)	1.05	0.9
C4MW7-cyclic	3.7	cyclic	7 (61)	1.07	1.1
C4MW18-linear	3.7	linear	18 (158)	1.07	1.0
C4MW18-cyclic	3.7	cyclic	18 (158)	1.08	0.9
C4MW25-linear	3.7	linear	24.5 (221)	1.05	0.8
C4MW25-cyclic	3.7	cyclic	24.5 (221)	1.07	1.2
C9MW7-linear	9.2	linear	7 (61)	1.05	1.1
C9MW7-cyclic	9.2	cyclic	7 (61)	1.07	1.0
C9MW18-linear	9.2	linear	18 (158)	1.07	1.0
C9MW18-cyclic	9.2	cyclic	18 (158)	1.08	1.0
C9MW25-linear	9.2	linear	24.5 (221)	1.05	1.0
C9MW25-cyclic	9.2	cyclic	24.5 (221)	1.07	0.8

a The iron oxide core diameter was determined by TEM. The Mn and PDI were determined by GPC on the individual grafted linear polymer chains. The grafting density of these chains was determined by TGA using the core diameter and Mn.

### 2.7 Intramolecular Click Reaction of N_3_-Terminated Poly(2-isopropyl-2-oxazoline)

For the cyclization of the polymer, two mixtures were prepared. The first mixture contained 0.1 mmol of the N_3_-terminated polymer in 567 mL of water previously deoxygenized in a nitrogen atmosphere. The second mixture contained 0.3 mmol of CuBr and 0.4 mmol of sodium ascorbate as the catalyst system dissolved in 2500 mL of water (previously deoxygenized in a nitrogen atmosphere). The polymer solution was dripped slowly into the catalyst solution under a nitrogen atmosphere. The reaction was stirred for 1 week. The product was purified by dialysis (cutoff: 3.5 kDa) for 3 days after the water was removed via freeze-drying. ^1^H NMR (300 MHz; CDCl_3_) δH: 7.63 (s, 1H), 3.48 (d, 2nH), 2.93–2.68 (d, 1nH), 1.12 (s, 6nH) (Figure S6). The successful cyclization was further supported by a shift to lower equivalent polystyrene molecular weight in SEC and changes to the N=N and N–N vibrational bands of the cyclic poly(2-isopropyl-2-oxazoline) compared to their linear equivalents (cf. Figures S4–S5 and S8).

### 2.8 Modification of Linear and Cyclic Poly(2-isopropyl-2-oxazoline)

The reaction of 24.5 kg mol^–1^ polymer is described as a representative sample. The functionalization of the polymer was prepared according to Kurzhals et al.^10^ Three grams (0.12 mmol, 1 equiv) of polymer was dissolved in 12 mL of dry chloroform. Then, 120.1 mg (1.2 mmol, 10 equiv) of succinic anhydride and 47.1 mg (0.38 mmol, 3.2 equiv) of DMAP (for the cyclic polymer 33.5 μL, 0.24 mmol, 2 equiv of TEA) were added and refluxed at 70 °C. After 48 h, the polymer was purified by extraction against water (twice 50 mL) and dried over Na_2_SO_4_. The dry polymer was dissolved in 25 mL of anhydrous DMF under a nitrogen atmosphere. COMU (132.3 mg, 0.31 mmol, 2.6 equiv) and 163 μL (0.94 mmol, 7.8 equiv) of DIPEA were added and stirred for 30 min. NDA (117.2 mg, 0.40 mmol, 3.3 equiv) was dissolved in 2 mL of anhydrous DMF, slowly added, and stirred for 48 h. The nitrocatechol-terminated polymer was precipitated in an Et_2_O–hexane mixture (ratio 1:1). The excess of 6-nitrodopamine was removed by dialysis (cutoff: 3.5 kDa) for 5 days. ^1^H NMR (300 MHz; CD_3_OD) δH: 7.53 (s, 1H), 6.72 (s, 1H), 4.28 (s, 4H), 3.53 (d, 4nH), 3.03–2.77 (d, 1nH), 1.11 (s, 6nH) (Figure S7).

### 2.9 Ligand Exchange on SPION (“Grafting to” of PiPrOx)

The grafting of the 24.5 kg mol^–1^ cyclic PiPrOx to the 3.7 nm particles (C4MW25-cyclic) is presented as a representative synthesis. Wet oleic acid-stabilized SPIONs (25.4 mg, inorganic fraction 28%) were dissolved in 1 mL of toluene. Five hundred milligrams of nitrocatechol-terminated PiPrOx was dissolved in 4 mL of DMF. The polymer solution was slowly dropped into the particle solution and reacted under ultrasonication for 4 days while keeping the temperature below 30 °C. The particles were precipitated in an Et_2_O–hexane mixture (ratio 1:1) and dialyzed against water (cutoff: 1000 kDa) for 4 days. Yield: 86 mg.

### 2.10 Methods

Transmission electron microscopy (TEM) images were recorded with an FEI Tecnai G2 (FEI Europe B.V., Austria) with 160 kV acceleration voltage using carbon grids. We calculated the size distributions of the iron oxide nanoparticle batches with the freeware Pebbles^45^ based on the analysis of >600 nanoparticles per batch. ^1^H NMR spectra were recorded on a BRUKER AV III 300 MHz spectrometer (Bruker Austria GmbH, Vienna, Austria). Chemical shifts were recorded in ppm. Residual protonated solvents CDCl_3_ (7.26 ppm), DMSO-*d*_6_ (2.50 ppm), and CD_3_OD (3.31 ppm) were used as a reference. The molecular weights of the polymers were measured by gel permeation chromatography (GPC) using a Malvern Viscotek GPCmax system (Malvern Instruments Ltd., Worcestershire, UK). The setup holds three MZ Gel SDSPlus columns (a precolumn followed by two columns with separation ranges of 10–2000 kDa and 1–40 kDa, respectively). A Kauer Smartline RI detector 2300 was used. DMF with 0.05 M LiBr was used as eluent with a flow rate of 0.5 mL min^–1^. A 3 g L^–1^ concentrated sample (50 μL) was injected and measured at 60 °C. The program OmniSEC 5.12 was used for data analysis. The system was calibrated with polystyrene standards (10 standards: from 1.5 to 4410 kg mol^–1^). GPC was also used to show the successful cyclization of the linear polymers by recording a shift to an elution profile corresponding to a lower effective Mn. A cyclic polymer has a lower hydrodynamic radius than the equivalent linear coil (Figure S5). The thermal gravimetric analysis (TGA) was performed on a Mettler Toledo TGA/DSC (Mettler Toledo GmbH, Vienna, Austria), with flow rates of 80 mL min^–1^ synthetic air (reactive gas) and 20 mL min^–1^ nitrogen (protective gas). The measured temperature range was 25 to 650 °C, with a heating rate of 10 K min^–1^. We used the data measured between 180 and 550 °C for subsequent calculations (Figure S9 and Figure S10). With the results from TGA, GPC (for the linear polymer and the corresponding linear polymers used for the cyclized polymers), and TEM, the grafting density (σ) was calculated using the formula
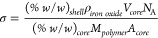
where (% *w*/*w*)_*shell*_ was the percentage of mass loss in TGA for the organic fraction corresponding to the polymer grafted onto the iron oxide core, *ρ*_*iron oxide*_ was the density of iron oxide, and *N*_*A*_ was the Avogadro constant. *V*_*core*_ was the volume, and *A*_*core*_ was the surface area of the iron oxide core calculated with the average diameters determined by TEM. *M*_*polymer*_ was the molecular weight of the polymer measured with the GPC, and (% *w*/*w*)_*core*_ was the residual mass percentage of the inorganic fraction in TGA. The results of the TGA measurements and calculations of the grafting densities are presented in Table S1. Dynamic light scattering (DLS) measurements of the critical flocculation temperature (CFT) and temperature cycling experiments were performed in Milli-Q water on a Malvern Zetasizer Nano-ZS (Malvern Instruments Ltd., Worcestershire, UK). Mean values and standard deviations of the count rate were calculated from three measurements for each temperature point. DLS was measured in the temperature range 25 to 50 °C with 1 °C steps except for the 7 kg mol^–1^ free polymer for which the temperature range was 25 to 70 °C. After each temperature change, the sample was equilibrated for 2 min and then measured three times. The CFT was determined by the onset of the increasing curve of count rate versus temperature. Microdifferential scanning calorimetry (DSC) measurements of core–shell particle dispersions and polymer solutions were performed in water with a Malvern MicroCal PEAQ-DSC (Malvern Instruments Ltd., Worcestershire, UK). The temperature range was fixed for all samples at 20 to 80 °C and a heating rate of 1 K min^–1^. Data processing was performed with the MicroCal PEAQ-DSC software version 1.51. The critical solution temperature (CST) was determined by the temperature at the onset of the corresponding specific heat capacity peak. The transition enthalpies per monomer unit were calculated using the following formula
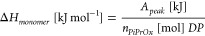
where Δ*H*_*monomer*_ was the average transition enthalpy per monomer unit, *A*_*peak*_ corresponded to the integral of the peak of the specific heat capacity curve measured with the DSC, *n*_*PiPrOx*_ was the molarity of the polymer, and *DP* was the degree of polymerization (number of monomer units in the polymer).

## 3 Results and Discussion

### 3.1 Preparation and Characterization of the Core–Shell Nanoparticles

Thermoresponsive core–shell nanoparticles were produced starting from monodisperse iron oxide nanoparticle cores (*cf*. Figure S1 TEM of cores and size distributions in the Supporting Information), synthesized by the heat-up method from the iron pentacarbonyl precursor in the presence of oleic acid pioneered by Hyeon et al.^42^ The “grafting to” method was used for grafting polymer shells of both cyclic and linear PiPrOx, respectively, to the cores by ligand replacement following previously published protocols.^8,10,41^ The polymers were synthesized and cyclized, as described in the Materials and Methods section. “Grafting to” enables the complete characterization of the polymers before grafting them to the inorganic cores, and it ensures monodisperse and homogeneously distributed grafts.

A disadvantage of the “grafting to” method is that polymers with high molecular weights are difficult to graft densely. A dense polymer brush is required for high colloidal stability and reversibility of thermoresponsive core–shell nanoparticle dispersions.^28^ Dense grafting requires a high-affinity and thermally stable anchor chemistry binding the polymer to the core. We choose 6-nitrodopamine (NDA), which complexes strongly to ferric compounds,^46−48^ as NDA was previously used to produce dense and thermally stable polymer grafts on iron oxide nanoparticles coated with oleate via ligand replacement.^40,49^

We prepared monodisperse core–shell nanoparticles with two different core diameters (3.7 and 9.2 nm) grafted with poly(2-isopropyl-2-oxazoline) of three different molecular weights (7 kg mol^–1^, 18 kg mol^–1^, and 24.5 kg mol^–1^) and with two different topologies (linear and cyclic), as reported in Table 1. The molecular weights were determined for the linear polymers using GPC and polystyrene as the calibration standard. This equivalent molecular weight is reported also for the cyclized polymers, which after cyclization show a shift in the GPC elution profile to a lower equivalent molecular weight (Figure S5). This shift is expected because the joining of the chain ends decreases the hydrodynamic size of the polymer coil, but it does not imply an actual reduction in Mn. A shoulder toward higher Mn is observed for some cyclic GPC elution profiles, which could be indicative of either increased chain–chain interactions or a minor fraction of linear polymer.

**Figure 1:**
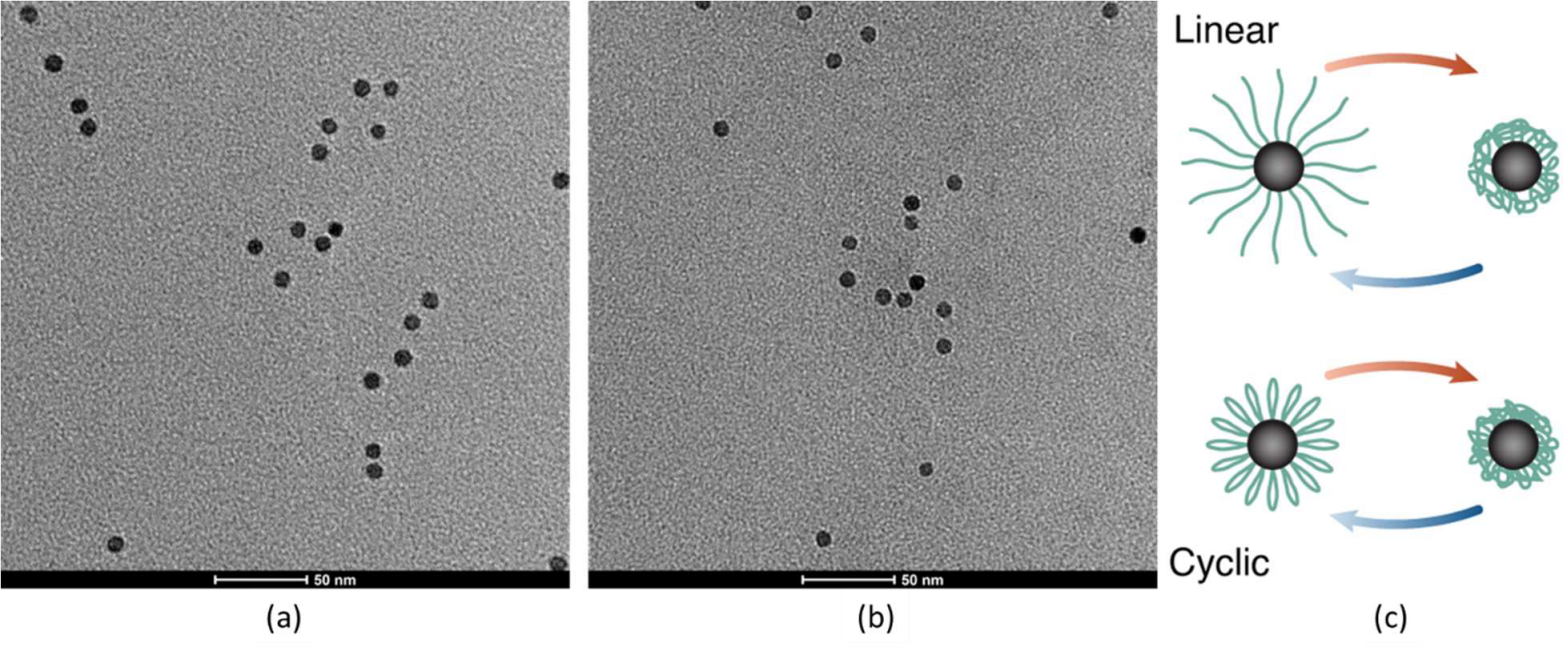
Transmission electron micrographs of 9.2 nm cores with grafted 18 kg mol^–1^ PiPrOx shells with (a) linear and (b) cyclic topography. The shells are not visible due to a lack of contrast in TEM. (c) Schematic of the nanoparticle cores functionalized with linear and cyclic polymer brushes, respectively, and the difference in shell structure upon heating and cooling.

The grafting densities were similar at ∼1 chain nm^–2^ to ensure both high colloidal stability^39,41^ and that we could directly compare all samples concerning the influence of polymer topology, molecular weight, and core size. These high grafting densities were achieved through our optimized ligand exchange protocols that favor the displacement in DMF of oleic acid by the NDA-functionalized polymer binding irreversibly to the iron oxide core surface.^41^ The high grafting density was achieved because the high curvature of the small nanoparticle cores results in a higher free volume per grafted polymer chain than on planar surfaces^50,51^ and easier access to the grafting sites on the core surface. All particles dispersed spontaneously in water after freeze-drying and storage. Figure 1 shows representative transmission electron micrographs of linear and cyclic polymer-grafted nanoparticles dried on a carbon grid and a schematic of the nanoparticles functionalized with polymer brushes of the two different topologies.

### 3.2 Critical Flocculation Temperature and Critical Solution Temperature: Linear versus Cyclic Free PiPrOx

DLS temperature-cycling experiments were used to determine the critical flocculation temperature (CFT). We defined the CFT as the temperature at which the average hydrodynamic size of the particle dispersion started to increase through aggregation. The aggregation of the core–shell nanoparticles above the CFT leads to a stronger scattering of light and increases the count rate measured by the DLS detector. The backscattered light intensity increases as a power-law with size, making the DLS count rate the most sensitive measure of the onset of aggregation and the CFT. This technique is more sensitive than turbidity measurements as only the scattered light is collected. Thereby, we avoided the high background in turbidity measurements by monitoring the backscattered intensity. Rapid aggregation to large aggregates leads to sedimentation, upon which a decrease in the count rate will be observed as less sample is exposed to the laser beam. Thus, the CFT can be spotted either as a rapid increase or decrease in the count rate, depending on the formed aggregates’ size. Sedimentation is also often observed as the second phase of aggregation upon further temperature increase and longer incubation time.

Polymers that are thermoresponsive in water typically show a lower critical solution temperature (LCST) in their phase diagrams. An LCST is in particular observed for hydrogen-bonding, nonzwitterionic polymers used for nanoparticles in biomedical and biotechnological applications. For these polymers, such as PiPrOx, we expect to find a critical solution temperature dependent on concentration. As polymer molecular weight and grafting into polymer brushes changes the local concentration (and the end-group fraction), it is instructive to investigate the internal breaking of hydrogen bonds during the CST by differential scanning calorimetry (DSC). DSC measures the specific heat capacity as a function of temperature *via* heat transport in and out of the sample.

**Figure 2:**
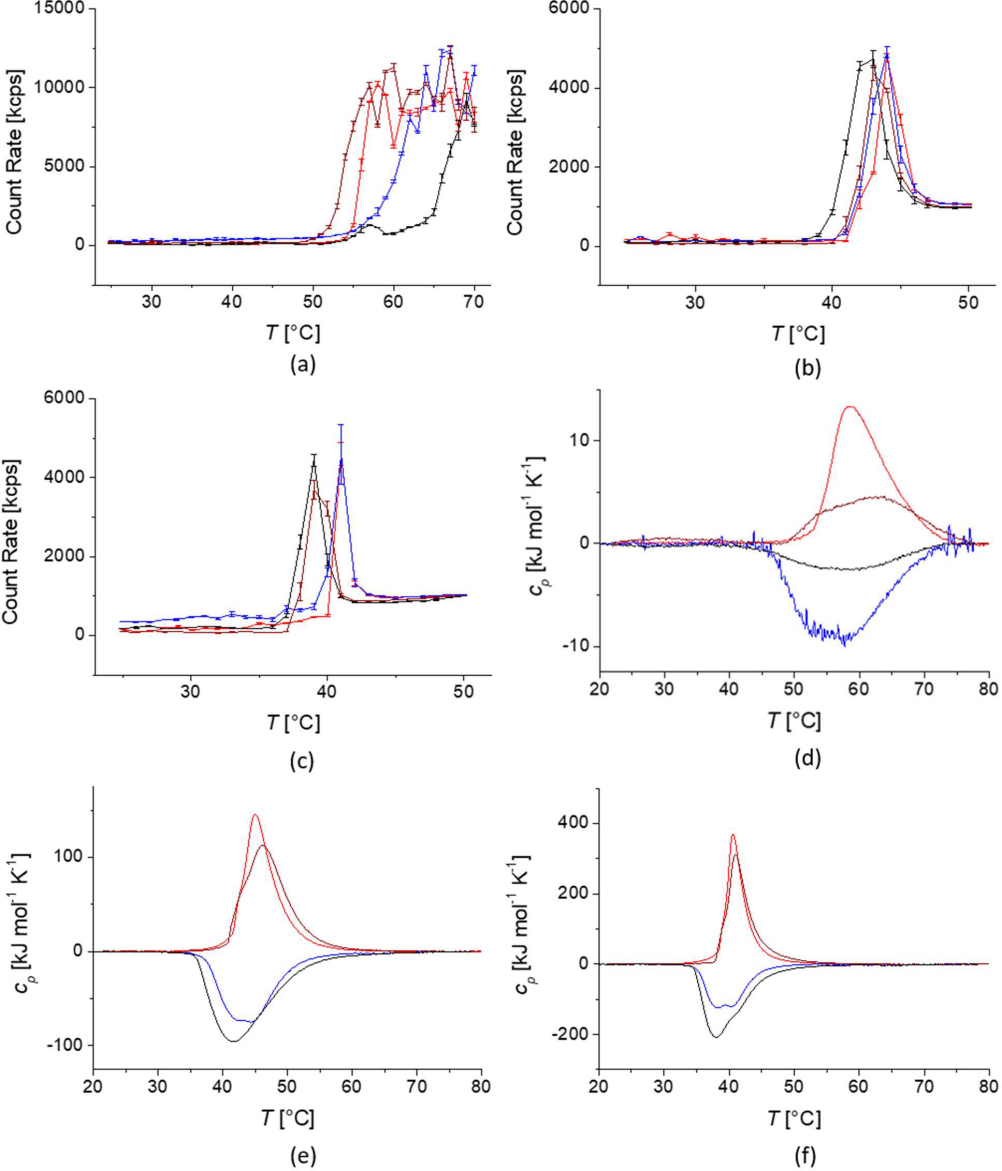
Temperature-cycled dynamic light scattering (DLS) for free PiPrOx (top): a) 7 kg mol^–1^, b) 18 kg mol^–1^, and c) 24.5 kg mol^–1^. Differential scanning calorimetry (DSC) for free polymer (bottom): d) 7 kg mol^–1^, e) 18 kg mol^–1^, and f) 24.5 kg mol^–1^. All samples refer to 1 g L^–1^ PiPrOx dispersed in Milli-Q water. Heating curves of linear polymer in red color, cooling curves of linear polymer in blue color. Heating curves of cyclic polymer in dark red color, cooling curves of cyclic polymer in black color. Mean values and standard deviations of the count rate were calculated from three measurements for each temperature point.

We first demonstrated the qualitative aspects of combining these two approaches to analyze the pure polymer samples at a concentration of 1 g L^–1^ in Milli-Q water in Figure 2. We observed distinct transitions for all polymers both by DLS and by DSC. The DSC indicated a small but significant shift in the peak *c*_*p*_ temperature (cf. Table 2, column 3, CST peak values) as well as a smaller peak area for cyclic compared to linear PiPrOx. The latter corresponds to the transition enthalpy and is proportional to the number of hydrogen bonds that break during the transition and their respective strengths. However, there was little difference in the onset temperature of the peak, and the differences between the topologically different polymers and the observed differences decreased with increasing molecular weight. Most cyclic polymers showed a larger hysteresis between heating and cooling CST compared to the linear polymers. Presumably, the formation of internal hydrogen bonds within the polymers contributed to this effect. The balance between water hydrogen bonds, intrapolymer hydrogen bonds, water, and chain entropy is different between cyclic and linear polymers, which makes a difference not only in CST but also in CST hysteresis expected.

**Table 2:** Characterization of Free PiPrOx Linear- and Cyclic-PiPrOx-Grafted Iron Oxide Nanoparticles during the Temperature-Cycling Measurements at 1 g L^–1^ in Milli-Q Water Using Both DLS and DSC^a^

		CST [°C]				
sample	CFT [°C]	onset	peak	Δ*H*_*monomer,heating*_ [kJ mol^–1^]	Δ*H*_*monomer,cooling*_ [kJ mol^–1^]	|| [%]
free polymer MW7-linear	52	48.3	58.6	2.25	–2.36	95
free polymer MW7-cyclic	49	47.6	62.3	1.25	–0.78	160
free polymer MW18-linear	41	37.4	45.0	5.68	–4.86	117
free polymer MW18-cyclic	40	38.2	46.1	5.86	–6.77	87
free polymer MW25-linear	39	35.1	40.6	6.24	–4.11	152
free polymer MW25-cyclic	37	37.7	41.0	6.33	–7.06	90
C4MW7-linear	42	27.6	46.7/52.7	2.18	–1.04	210
C4MW7-cyclic	43	39.1	63.0	1.41	–0.41	344
C4MW18-linear	35	29.8	38.0	1.46	–1.35	108
C4MW18-cyclic	36	36.1	41.7	3.60	–4.30	84
C4MW25-linear	35	27.9	39.9/45.9	14.12	–4.42	319
C4MW25-cyclic	37	33.1	41.2	4.38	–3.19	137
C9MW7-linear	33	26.6	53.0	3.84	3.97	97
C9MW7-cyclic	31	24.2	64.4	1.76	–1.23	143
C9MW18-linear	36	33.6	40.9	4.32	–3.65	118
C9MW18-cyclic	39	33.8	43.6	3.23	–3.41	95
C9MW25-linear	36	31.5	40.1	5.07	–4.52	112
C9MW25-cyclic	38	32.9	40.6	5.02	–3.25	154

a All values are extracted from the heating curves.

The DLS measurements of the global transition of the polymer solution showed more distinct differences in the onset of the aggregation. Cyclic PiPrOx aggregated at a lower CFT than their linear equivalents. It is also noteworthy that the CSTs and CFTs decreased with increasing molecular weight for all polymers but that the hysteresis in the temperature-cycled transition was generally shifted to a higher temperature for the CFT while it is consistently lower for the CST. Interestingly, the difference between the onset of the transition in DSC and the CFT was always drastically smaller for cyclic polymers than for linear polymers, indicating that once dehydration starts (at a higher temperature), aggregation proceeds more easily (at a lower temperature). One interpretation of these differences is that the hydration of the cyclic PiPrOx is weaker due to its topologically confined conformation and smaller hydrated size, leading to a lower transition enthalpy and earlier onset of aggregation. A lower enthalpy for the LCST transition of cyclic polymers has previously been described for PNIPAM by Winnik et al.; however, in contrast to our study, they reported a small increase in LCST for cyclic PNIPAM.^52^ A huge increase in LCST for cyclic compared to linear PiPrOx was reported recently by Jung et al.,^53^ while Liu and co-workers similar to us reported a lower LCST for cyclic PNIPAM compared to linear PNIPAM.^54^ It is not clear why such qualitatively different behaviors were observed comparing our and Liu’s studies on the one side and Winnik and Jung’s on the other side. Still, the LCST is also sensitive to molecular weight, end-group chemistry, and polymer concentration. Jung et al. investigated polymers with molecular weights lower than 4 kg mol^–1^ at higher concentrations using turbidity measurements instead of DLS,^53^ which might have resulted in the different phase diagram.

### 3.3 Critical Flocculation Temperature for Linear and Cyclic PiPrOx-Grafted Nanoparticles

**Figure 3:**
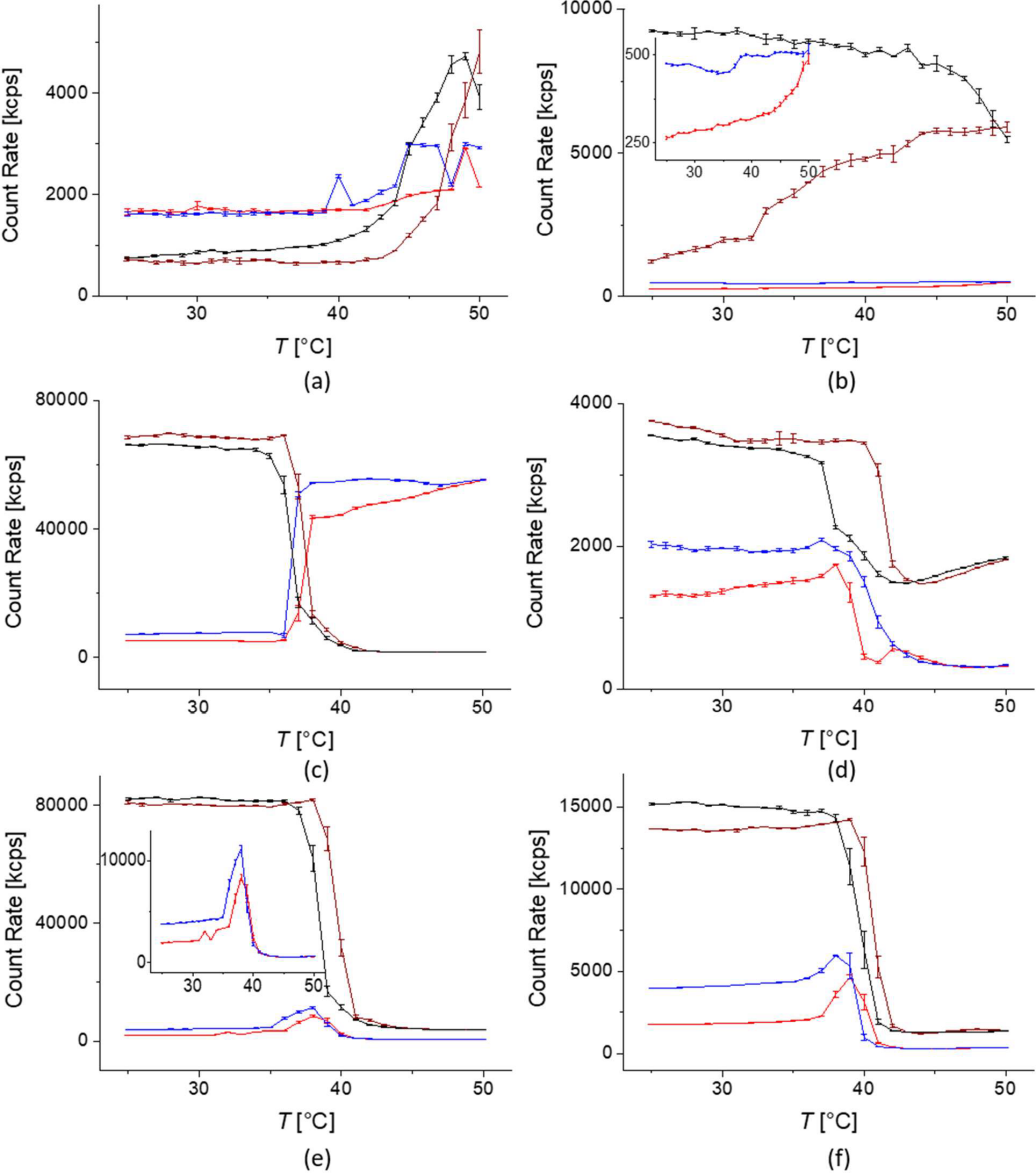
Temperature-cycled dynamic light scattering data for 3.7 nm core size PiPrOx-grafted core–shell nanoparticles (left) and 9.2 nm core size core–shell nanoparticles (right) dispersed in Milli-Q water at a concentration of 1 g L^–1^ for: 7 kg mol^–1^ PiPrOx (a and b), 18 kg mol^–1^ PiPrOx (c and d), and 24.5 kg mol^–1^ PiPrOx (e and f). Heating curves of linear polymer in red color, cooling curves of linear polymer in blue color. Heating curves of cyclic polymer in dark red color, cooling curve of cyclic polymer in black color. Mean values and standard deviations of the count rate were calculated from three measurements for each temperature point.

Figure 3 shows the average count rate of the PiPrOx-grafted iron oxide nanoparticle dispersions against the temperature measured by DLS upon heating and cooling at a polymer concentration of 1 g L^–1^ in Milli-Q water. It reveals reversible aggregation and mostly followed by sedimentation for all core sizes, polymer architectures, and molecular weights. Table S2 shows the comparison between the hydrodynamic diameter before and after the temperature cycle experiments, as well as the CFTs extracted from the data in Figure 3. The CFT was chosen as the onset of the transition in the DLS count rate curve.

**Figure 4:**
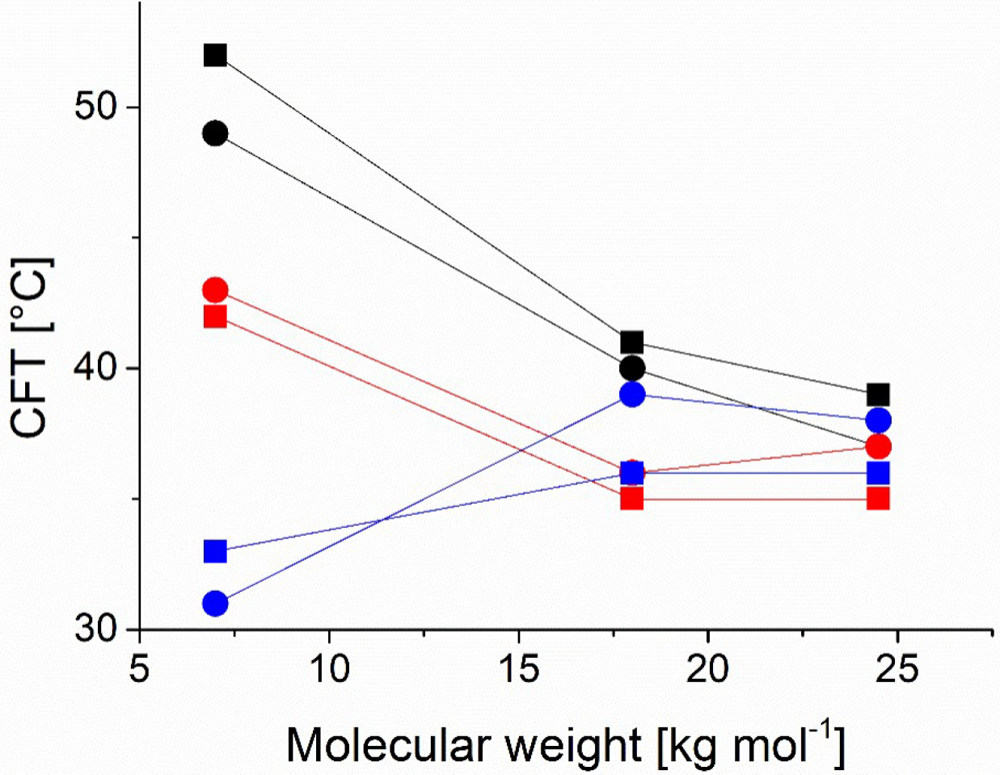
Summary of the critical flocculation temperature as a function of molecular weight of cyclic and linear PiPrOx for free polymer and grafted on iron oxide nanoparticles, measured in Milli-Q water at a concentration of 1 g L^–1^ by DLS. The CFT is chosen as the onset of the transition in the DLS count rate curve. Black: free polymer, red: 3.7 nm core, blue: 9.2 nm cores, squares: linear polymer, circles: cyclic polymer.

Interestingly, from the data from Figure 3 and Table 2 summarized in Figure 4, we observed slightly higher CFTs for cyclic polymer brush shells than for their linear counterparts grafted at similar densities to the nanoparticles. This contrasts the results for the free polymers, which showed the opposite behavior, but it agrees qualitatively with our previous observations for low molecular weight PiPrOx grafted very densely to large cores.^37^ There is one exception in the data set. The 7 kg mol^–1^ PiPrOx grafted to the 9.2 nm cores revealed a CFT that decreased strongly for both linear and cyclic polymers compared to all other samples, with the largest decrease for the cyclic polymer brush shell.

The colloidal stability of core–shell nanoparticles results from the superposition of the repulsive osmotic potential of the polymer brush shell and the attractive van der Waals potential of the core.^28,55^ As the particle size increases, the curvature decreases, which leads to a thicker brush.^50^ However, the attractive van der Waals potential increases more drastically (linearly) with size.^55^ In a system where these forces are balanced, the loss of screening of the core–core attraction might drive the aggregation earlier than when the flocculation is driven mainly by shell–shell interaction caused by the lowering of polymer solubility.

We interpret our results such that the outlier for the thinnest polymer shells and the largest cores shows the effect of core–core interaction on the CFT. In this case, the onset of the thermal transition, leading to a thinner shell, could trigger aggregation before the full solubility transition of the shell. The repulsion of the polymer shell was sufficient to prevent aggregation of the smaller core diameter nanoparticles as their vdW attraction was weaker, and the higher molecular weight polymer shells are thicker and denser. However, for the large cores, the significant contribution of the core vdW potential over a distance presumably exceeding that of the collapsed shell led to a drastic lowering of the CFT (Figure 4), explaining the opposite dependence of the CFT on polymer Mw compared to the other samples. As the polymer Mw was increased, the shell thickness increased, which reduced the relative importance of the core–core attraction for setting the CFT.

Further supporting our interpretation of the effect of superposing the attractive core potential on the brush repulsion is the irreversible aggregation occurring for the large cores grafted with either linear or cyclic 7 kg mol^–1^ PiPrOx. Once the cores came sufficiently close after the collapse of the polymer shell to fall into a vdW minimum in the interaction potential, rehydration of the shell upon reducing the temperature was no longer sufficient to disaggregate the cores.

Cyclic polymers have a lower free volume per monomer and conformational entropy than linear polymers, suggesting a lower CFT as free polymers.^56^ Additionally, a cyclic polymer brush is thinner than a linear polymer brush if the polymers have the same molecular weight and grafting density.^32^ These facts imply that we should expect lower stability of nanoparticle dispersions stabilized with cyclic than with linear polymer brushes. Thus, the explanation for why the cyclic polymer brushes made the nanoparticles more colloidally stable to the thermally induced solubility transition must be found in the structure of the brushes.

A cyclic brush will have a higher segment density close to the core than a linear brush. Again, this should lead to a lower CST and CFT, as we earlier found for the dependence of the CFT and CST on the segment density profile of linear brush core–shell nanoparticles.^8,13^ However, the entropy-driven aggregation could also be seen as depending on the reduction of polymer area in the system. Linear polymer brushes can interdigitate and reduce the internal area of the brush. Interdigitation of the shells brings the cores closer and can contribute an attractive core–core interaction to the CFT transition. Conceptually, one could consider cyclic polymer brushes to have twice the effective grafting density of the equivalent linear brush but with a lower effective molecular weight.^57^ Theoretically^58^ and experimentally on planar systems,^30^ their topology has been shown to prohibit brush interdigitation. Therefore, we expect the colloidal interactions of cyclic brush-stabilized nanoparticles to be similar to those of a dispersion of slightly lyophobic solid spheres when the polymer chains are not fully hydrated. The effective reduction in polymer dehydration or internal polymer surface area upon aggregation is lower in such a system. The internal area of the polymer shells cannot be reduced by interdigitation and conformal contact. Furthermore, the grafted cyclic polymers lack free end-segments, while the linear brush has free end-segments preferentially located in the outer part of the brush.^50^ End-segments have a large influence on the CST of thermoresponsive polymers, especially for low-molecular-weight polymers.^59^ We hypothesize that the suppression of interdigitation is the main reason for the reversed dependence on cyclization for the CFT for free polymers vs linear polymers. It dominates over the traditionally acknowledged contributions that predict a lower CFT for cyclic polymer brush-stabilized nanoparticles. However, we acknowledge that, e.g., Winnik and co-workers previously have discussed similar topological mechanisms (prohibition of intermolecular linking and absence of end groups) as leading to higher LCST also for free cyclic polymers than for their linear counterparts.^52^

Previous works by Yamamoto and co-workers comparing micelles of cyclic block copolymers and the stability of Au nanoparticles mixed with lyophilic cyclic polymers also showed increased colloidal and thermal stability when cyclic polymers instead of linear polymers were added.^34,35^ However, the topological effect in these cases could primarily be explained as increased polymer packing at the micelle or NP interface for cyclic polymers due to the topological constraints. The same effect could partly explain our previously published results showing higher grafting densities and colloidal stability for cyclic than for linear PiPrOx-grafted nanoparticles.^37^ In our current study, we held the grafting density equal for all cyclic and linear polymer brushes. Hence, our observations are exclusively due to a difference in nanoparticle shell structure, not grafting density.

For both linear and cyclic PiPrOx-grafted nanoparticles, we observed a decrease in CFT with increasing molecular weight. It follows the general rule that higher molecular weight polymers have lower CSTs, which confers lower CFTs to core–shell nanoparticles stabilized with polymer brushes. It is interesting to note that this dependence decreased with increasing core size (*cf*. Figure 4). We expected the decreasing slope in Figure 4 with the polymer molecular weight. A lower fraction of the polymer experiences a high local segment concentration close to the core’s surface for a high than for a low Mw polymer.^8^ Conversely, a greater fraction experiences the average segment density in the free coil-like part of the polymer in the outer parts of the spherical brush.^13^ The concentration in the interior of a polymer coil does not scale strongly enough with molecular weight to compensate for the reduced importance of the inner part of the shell.^50,60,61^ We similarly expect a reduced dependence of the CFT on molecular weight when the polymer is grafted to a surface and when the surface curvature decreases. There are too few data points to confirm this conclusion, but the existing data points indicate that this is the case for all polymer brushes.

### 3.4 Critical Solution Temperature Transitions within the Shell for Linear and Cyclic PiPrOx-Grafted Nanoparticles

Besides the CFT, an important characteristic of the samples is the local change in the polymer chains’ solubility, described by the critical solution temperature transition. As described above, the polymer solubility transition is the origin of the CFT by shifting the balance of the colloidal interactions of the core–shell nanoparticle. However, the local nanoparticle environment and imposed morphology influence the CST transition of grafted polymers. Thereby, a multifaceted phase behavior can be observed for the CST transition.^9,28^ A CST transition occurs over a broad temperature range, but it is cooperative. It can split into multiple distinct transitions in response to differences in the local environment of the polymer.^9,11,50,62^ The solubility transition includes the breaking of hydrogen bonds of water molecules to the polymer in favor of the higher entropy of bulk water. Thus, we can map the transition by differential scanning calorimetry (DSC). DSC measures the heat required to keep the sample at the same temperature as the pure continuous phase during a temperature scan. Each step of a temperature-induced transition can be observed by the change in specific heat capacity due to the breaking or forming of bonds. By integrating the data, we can determine the enthalpy of the transition. The CFT does not have to correspond to the onset of a polymer’s solubility transition, nor must it correspond to the temperature at the peak value, which is mostly referred to as the CST in a DSC measurement. However, we expect correlations between these transitions due to the change in the nanoparticles’ interaction potential as a function of polymer hydration and shell thickness.

**Figure 5:**
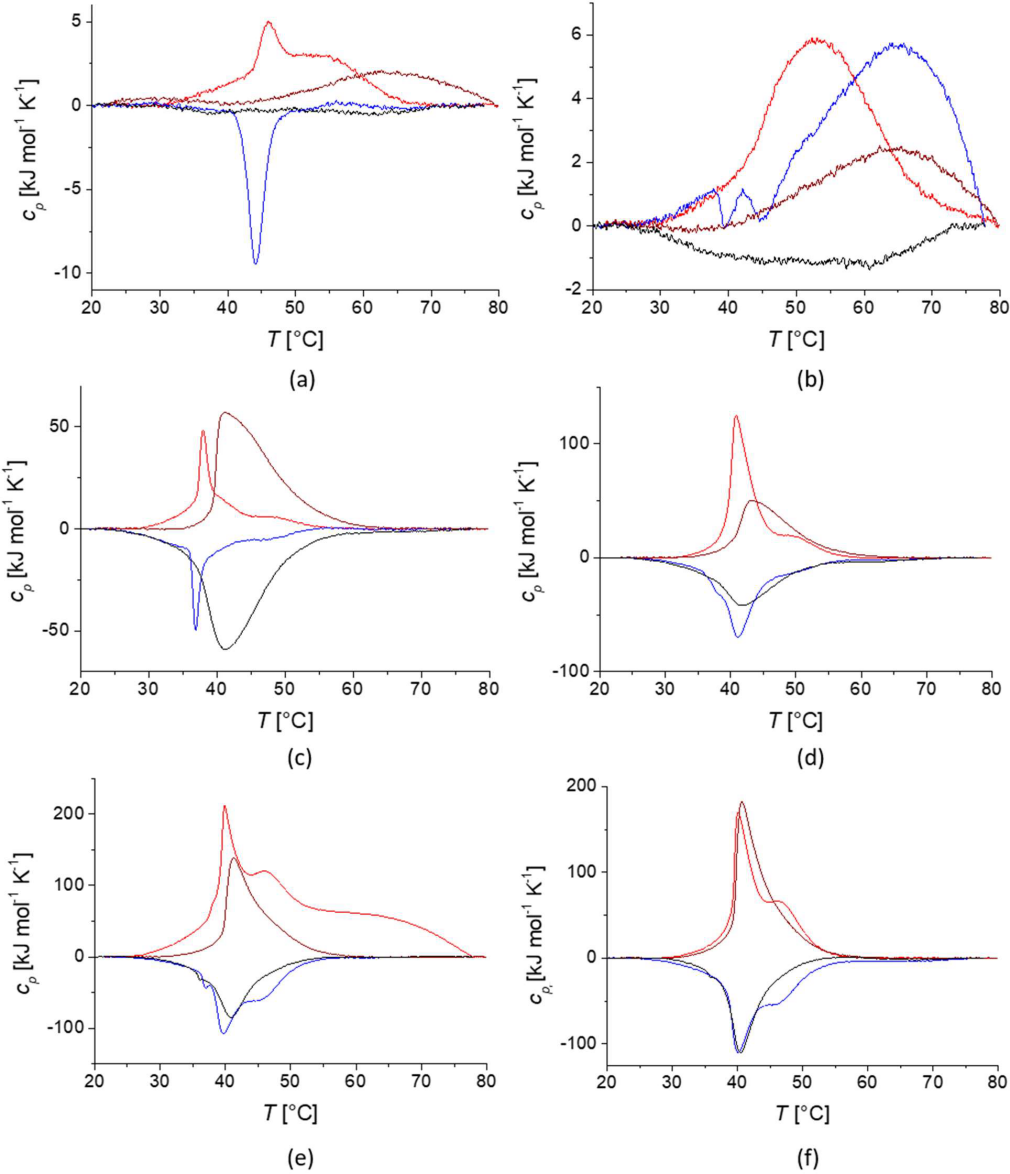
Differential scanning calorimetry data for 3.7 nm core size PiPrOx-grafted core–shell nanoparticles (left) and 9.2 nm core size core–shell nanoparticles (right) dispersed in Milli-Q water at a concentration of 1 g L^–1^ for: 7 kg mol^–1^ PiPrOx (a and b); 18 kg mol^–1^ PiPrOx (c and d); 24.5 kg mol^–1^ PiPrOx (e and f). Heating curves of linear polymer in red color, cooling curves of linear polymer in blue color. Heating curves of cyclic polymer in dark red color, cooling curves of cyclic polymer in black color.

Figure 5 shows the DSC data for the heating and cooling traces of the PiPrOx-grafted iron oxide nanoparticle dispersions. The DSC curves of the core–shell nanoparticles were very different from those of the free polymers shown in Figure 2. Especially the linear polymer brush shells showed more complex behaviors. The transition peaks were substantially broader but additionally seemed comprised of multiple, distinguishable but convoluted transitions. For the large 9.2 nm cores, two convoluted peaks seemed to be present for at least the two higher molecular weight polymers, while three convoluted peaks were observed for the 4 nm cores. We previously reported this behavior for densely grafted linear polymer brushes on nanoparticles^8,13^ and attributed it to the effect of the high curvature imposed on the brush by the grafting to the surface of small nanoparticles. Tentatively, the different peaks correspond to different scaling regimes of the brush in analogy with the star polymer model for polymer-grafted core–shell nanoparticles,^50,57,63−65^ which was experimentally verified for highly grafted nanoparticles.^51^ The lowest temperature peak corresponds to the onset of the brush transition, which takes place in the part of the shell closest to the grafting point to the iron oxide surface with the highest polymer segment density. The second peak corresponds to the intermediate part of the shell, which sees a polynomial drop in segment density. The highest temperature transition corresponds to the outer part of the shell, similar in density to a mushroom or free coil conformation.^8,13^ Similar results were also reported by Shan et al. for PNIPAM grafted onto very small gold particles.^9^

Interestingly, the cyclic polymer brushes did not show the same behavior in DSC as the linear ones. While the peak widths changed after grafting the cyclic PiPrOx onto the nanoparticles, there was no clear indication of multiple distinguishable transition peaks. The transitions had considerable similarities with the corresponding peaks for free cyclic PiPrOx. Compared to the corresponding free polymers, the CSTs of the cyclic polymer brushes were lower. A comparison of the cyclic brush-stabilized nanoparticles to the reference systems suggested that the cyclic topology led to a brush shell of uniform high polymer segment density, which transitioned cooperatively. Again, this indicates that grafting to the nanoparticle surface changes the LCST transition of cyclic polymers differently than for linear polymers. Free cyclic polymers were reported to have broader transitions and show less cooperativity than linear thermoresponsive polymers.^52,54^ The cyclic constraints yielding a denser and more uniform brush on curved surfaces conserves a uniform LCST transition, while the linear polymer restructures in a concave brush and displays a broadening of the transition.

**Figure 6:**
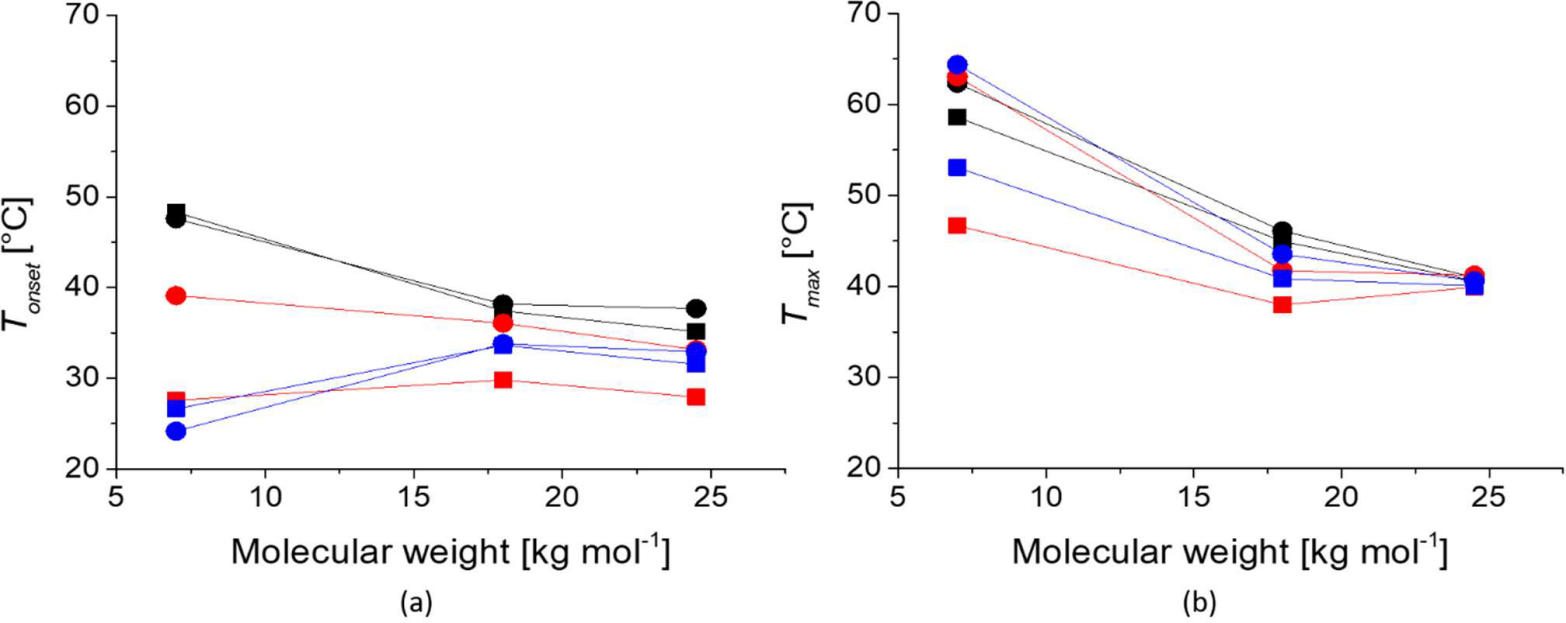
Summary of the critical solution temperatures as a function of molecular weight of cyclic and linear PiPrOx for free polymer and grafted on iron oxide nanoparticles, measured in Milli-Q water at a concentration of 1 g L^–1^ by DSC. a) The CST was chosen as the temperature at which *c*_*p*_ starts increasing, *T_onset_*, and b) the CST was chosen as the temperature at the maximum in *c*_*p*_ (corresponding to the first local maximum in the curve), *T_max_*. All values are extracted from the heating curves. Black: free polymer, red: 3.7 nm core, blue: 9.2 nm cores, squares: linear polymer, circles: cyclic polymer.

Figure 6 shows the CSTs for all samples chosen in two different ways, i.e., as the temperature at the onset of the increase in *c*_*p*_ (Figure 6a) and as the temperature at the peak of the *c*_*p*_ curve (Figure 6b). Table 2 additionally lists all the local peak temperatures fitted by the DSC software and compares the DLS and the DSC data. We observed that the onset temperature as the CST measure had the highest qualitative similarity with the CFT determined by DLS (*cf*. Figure 4). It predicted the much higher CFTs for the free coil polymers than for the nanoparticle-grafted polymers. It also captured the drop in transition temperature for the 9.2 nm cores grafted with 7 kg mol^–1^ PiPrOx. The latter strongly suggests that having a high fraction of the polymer at high local segment density imposed by grafting to the core and promoted by the lower curvature of larger cores caused a solubility and aggregation transition at low temperature. It additionally demonstrates that the lowering of the CFT for the 9.2 nm cores grafted with 7 kg mol^–1^ cyclic or linear PiPrOx also could have its origin traced to an earlier transition in the inner part of the shell. The dependence is in qualitative agreement, but we note that the CST determined by the onset of the DSC transition is much lower than the CFT determined by DLS.

Further, the CSTs set by both the highest peaks of the *c*_*p*_ curves, as well as the onset of the increase in the curves, was higher for the cyclic polymer brushes than for linear brushes (Figure 6 and Table 2). This is the reverse relationship to what was observed for the CFT (*cf*. Figure 4). The higher CST of the cyclic than the linear brushes could be surprising. The CST of the free coil polymer followed the expected trend, which was clearest for *T*_*max*_, namely that the denser and more restricted cyclic polymer had the lowest CST. In all cases, the CST defined by the onset was lower than the CFT. The two were even in fair agreement for the free coil polymers, while *T*_*onset*_ was significantly lower for grafted polymers than the CFT of those nanoparticles. Given that the grafting also always lowered *T*_*onset*_, we conclude that grafting affected cyclic polymers much less in terms of their solubility transition than it affected linear polymers. Tentatively, this could be because the topology has already imposed a conformational restriction affecting the transition on cyclic polymers. Therefore, a relatively larger conformational restriction was imposed on the linear than on the cyclic polymers when one end-segment was tethered to the particle core surface.

It is noteworthy in this context that the DSC peaks of linear and cyclic polymers had different shapes. The peak value is shifted slightly to higher *T* for cyclic polymers, and the full-width-at-half-maximum values are much higher for cyclic than for linear polymers if we compare the free polymers and the first peak of the grafted linear polymers to the single transition of the grafted cyclic polymers, respectively. Hence, tentatively, there seems to be an additional effect favoring the colloidal thermal stability of nanoparticles grafted with cyclic polymers. The CFT primarily correlated with the transition of the nanoparticle shell’s inner part, presumably due to the lower and shorter-range repulsive screening of the core–core attraction. Thus, the higher temperatures of the onset and peak of the transitions observed for grafted cyclic polymers led to the higher CFT of those nanoparticles.

It is difficult from the data set to determine how much higher stability stemmed from the slight shift to a higher transition temperature and how much came from the greater homogeneity and suppression of interdigitation of the cyclic shells. None of these CSTs quantitatively coincided with the CFT. The CFTs tended to lie between *T*_*onset*_ and *T*_*max*_. The differences between the three values decreased as the molecular weight increased.

### 3.5 Transition Enthalpies of Polymers with Different Architectures

**Figure 7:**
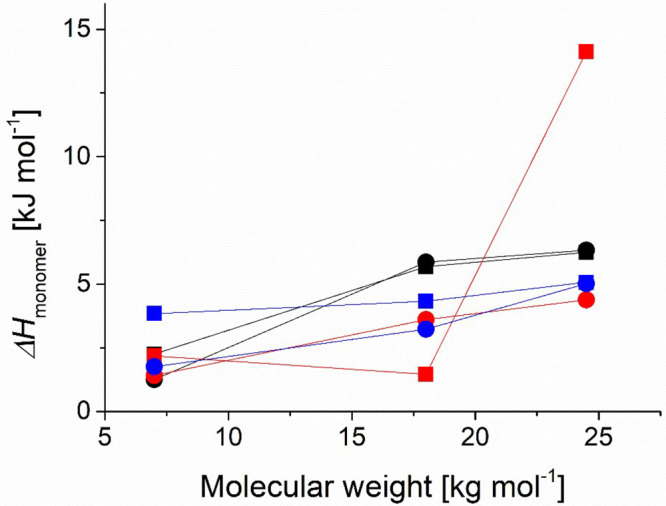
Average transition enthalpy per monomer for free polymer and polymers grafted to 3.7 nm cores and 9.2 nm cores, as a function of the molecular weight. Δ*H*_*monomer*_ was calculated from integrating the corresponding DSC heating curve and normalizing by the average degree of polymerization. Black: free polymer, red: 3.7 nm core, blue: 9.2 nm cores, squares: linear polymer, circles: cyclic polymer.

Finally, we used the DSC data to calculate the enthalpy per monomer of the complete solubility transition for free polymer, core–shell nanoparticles, and the respective polymer topology. The values measured for the heating trace are summarized in Table 2 and graphically shown in Figure 7. As in previous studies, the free polymers had higher transition enthalpies per monomer unit than the nanoparticle-grafted polymers.^8,13^ It follows the pattern that the transition enthalpy per monomer decreases with an increase of the average polymer segment density within the coil that, e.g., increases for higher molecular weight polymers. Grafting to the particle surface also forces a higher local segment density, which decreases the transition enthalpy. Interestingly, the free cyclic polymers did not have significantly lower transition enthalpy than their linear analogs, in contrast to reports for PNIPAM.^52^ At least for the higher molecular weights, the transition enthalpies were close to identical. The transition enthalpies for the nanoparticle-grafted polymers seem inconsistent for the linear polymers on the 3.7 nm cores. In contrast, the results for the free polymers and the 9.2 nm cores seem consistent with a lowering of the transition enthalpies due to grafting, which is tentatively larger for the cyclic polymers than for the linear polymers.

The comparison in Table 2 of the enthalpy per monomer unit between heating and cooling shows that the transitions were either similar or higher in the heating measurements. This indicates that not all polymer–water hydrogen bonds were reformed during the cooling and rehydration process after they broke during the heating. The large differences in transition enthalpy between heating and cooling for the 3.7 nm core samples and the 7 kg mol^–1^ PiPrOx indicate that the hydration of these samples was not fully reversible. The 7 kg mol^–1^ polymers had traces of residual CuBr that might have contributed to this variability.

The enthalpy derived from the cooling curves exceeded the heating transition enthalpies for some samples. A likely explanation would be that these samples might not have been in an equilibrated solvation state before the heating run. Our samples were freeze-dried at the end of the synthesis and redispersed in water before their thermal properties were investigated. This procedure could influence the starting hydration state. The rehydration of poly-2-alkyl-2-oxazolines can be slow and is not always fully reversible if internal hydrogen bonds are formed in place of those with water.^11,66,67^ In summary, we did not find a trend that distinguished the cyclic and linear polymers with respect to reversibility.

## 4 Conclusion

We have investigated the influence of varying the polymer topology from linear to polymer for the thermoresponsive properties of poly-2-isopropyl-2-oxazoline brush-stabilized nanoparticles. Both polymer topologies could provide thermoresponsiveness and colloidal stability, including reversible aggregation, when grafted as polymer brush shells on nanoparticles. However, we demonstrated that cyclic polymers increase the colloidal stability and increase the CFT of the colloidal aggregation when all other conditions are the same.

The increase in transition temperature, i.e., stability, is more pronounced for nanoparticles than for free polymers. We traced this to that linear polymer brush shells show multiple internal solvation transitions within the shell, while the cyclic polymers display one uniform transition. The number and strength of the brush’s solubility transitions were much less affected by the core size and surface curvature for cyclic than for linear polymers.

The colloidal aggregation was driven by the initial desolvation of the inner part of the stabilizing shell. This transition occurred at a lower temperature for the linear polymers than for the cyclic ones after grafting. The cyclic polymers are expected to interpenetrate less between opposing shells. We assume these differences in the shell structure and interparticle interaction explained the colloidal behavior due to the strong correlation with the observed behavior. Therefore, a higher colloidal stability observed for nanoparticles grafted with cyclic polymer brushes. We also showed that the grafting affects the properties more than the topology and that the core–core interaction should be taken into account to understand the colloidal aggregation for thin polymer brush shells.

In summary, while the examples of thermoresponsive cyclic polymers are few to date, our measurements show that topology is an additional way by which the colloidal interactions of polymer-functionalized nanoparticles can be efficiently tailored. Variation of the topology is particularly useful for applications in which a thin polymer shell is desired to keep the total particle size low.^32^

## Supporting Information Available

Figure S1, size distribution of iron oxide nanoparticles, measured with TEM and calculated with the freeware Pebbles; Figure S2, NMR of 2-azidoethylamine; Figure S3, NMR of 6-nitrodopamine; Figure S4, GPC of linear poly(2-isopropyl-2-oxazoline); Figure S5, GPC of cyclic poly(2-isopropyl-2-oxazoline); Figure S6, NMR of cyclic poly(2-isopropyl-2-oxazoline); Figure S7, NMR of 6-nitrodopamine modified linear and cyclic poly(2-isopropyl-2-oxazoline); Figure S8, ATR-FTIR spectra of linear (azide-terminated) poly(2-isopropyl-2-oxazoline) before cyclization and cyclic poly(2-isopropyl-2-oxazoline); Figures S9 and S10, TGA of PiPrOx-grafted core–shell nanoparticles; Table S1, TGA results for PiPrOx-grafted core–shell nanoparticles; and Table S2, comparison of hydrodynamic diameter of PiPrOx-grafted nanoparticles before and after temperature cycle and their CFT (PDF)

## Abbreviations

DMA: *N,N*-dimethylacetamide
TEA: triethylamine
DMAP: 4-(dimethylamino)pyridine
DIPEA: *N,N*-diisopropylethylamine
DMF: *N,N*-dimethylformamide
MeTs: methyl-*p*-tosylate
PrTs: propargyl *p*-toluenesulfonate
COMU: 1-[(1-(cyano-2-ethoxy-2-oxoethylidenaminooxy)dimethylaminomorpholino)]uronium hexafluorphosphate
KOH: potassium hydroxide
Et_2_O: diethyl ether
DCM: dichloromethane
CuBr: copper bromine
SPION: superparamagnetic iron oxide nanoparticle
PiPrOx: poly(2-isopropyl-2-oxazoline)
TEM: transmission electron microscope
GPC: gel permeation chromatography
TGA: thermal gravimetric analysis
DLS: dynamic light scattering
CFT: critical flocculation temperature
DSC: differential scanning calorimetry
CST: critical solution temperature
NDA: 6-nitrodopamine
PNIPAM: poly(*N*-isopropylacrylamide)
