# Supplementary material for: Thermoresponsive Nanoparticles with Cyclic-Polymer-Grafted Shells Are More Stable than with Linear-Polymer-Grafted Shells: Effect of Polymer Topology, Molecular Weight, and Core Size
Source: J Phys Chem B. 2021 Jun 22;125(25):7009–23. doi: 10.1021/acs.jpcb.1c00142 (PMC8279546; doi:10.1021/acs.jpcb.1c00142)
Supplement: Supplementary file 1 — jp1c00142_si_001.pdf [file jp1c00142_si_001.pdf]

Supporting information

Thermoresponsive Nanoparticles with Cyclic-Polymer-Grafted Shells Are More Stable than with Linear-Polymer-Grafted Shells: Effect of Polymer Topology, Molecular Weight, and Core Size

*Max Willinger and Erik Reimhult**

Institute for Biologically Inspired Materials, Department of Nanobiotechnology, University of Natural Resources and Life Sciences Vienna, Muthgasse 11, 1190 Vienna, Austria

* Email: erik.reimhult@boku.ac.at

Keywords: core-shell iron oxide nanoparticle, thermoresponsive, poly(2-isopropyl-2-oxazoline), cyclic polymer brush, polymer topology, differential scanning calorimetry, colloidal stability

ORCID: 0000-0001-6905-9623, 0000-0003-1417-5576

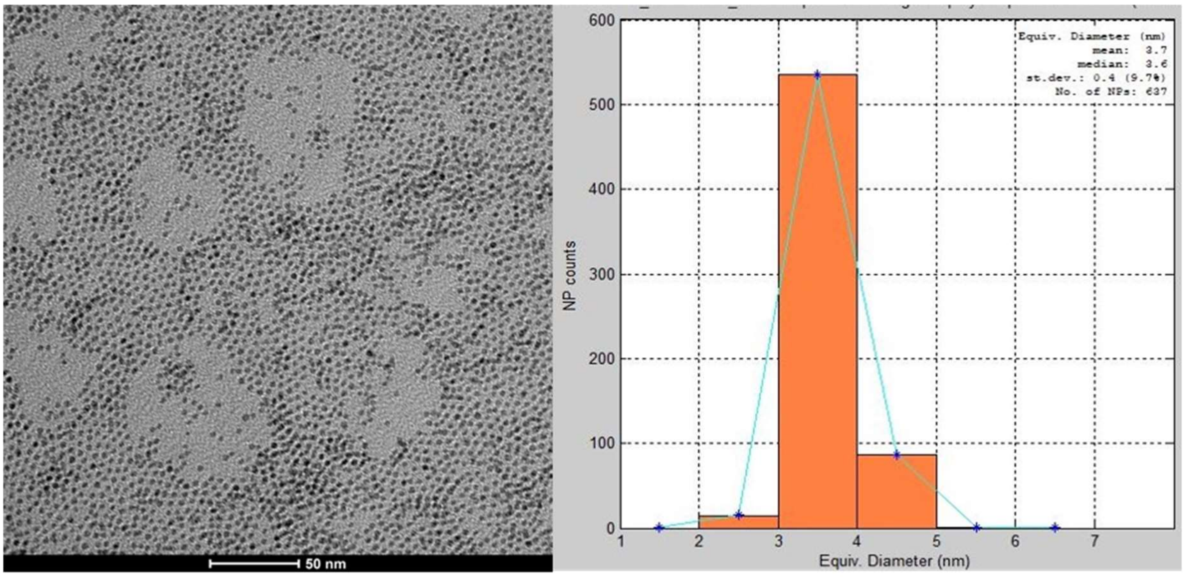

(a)

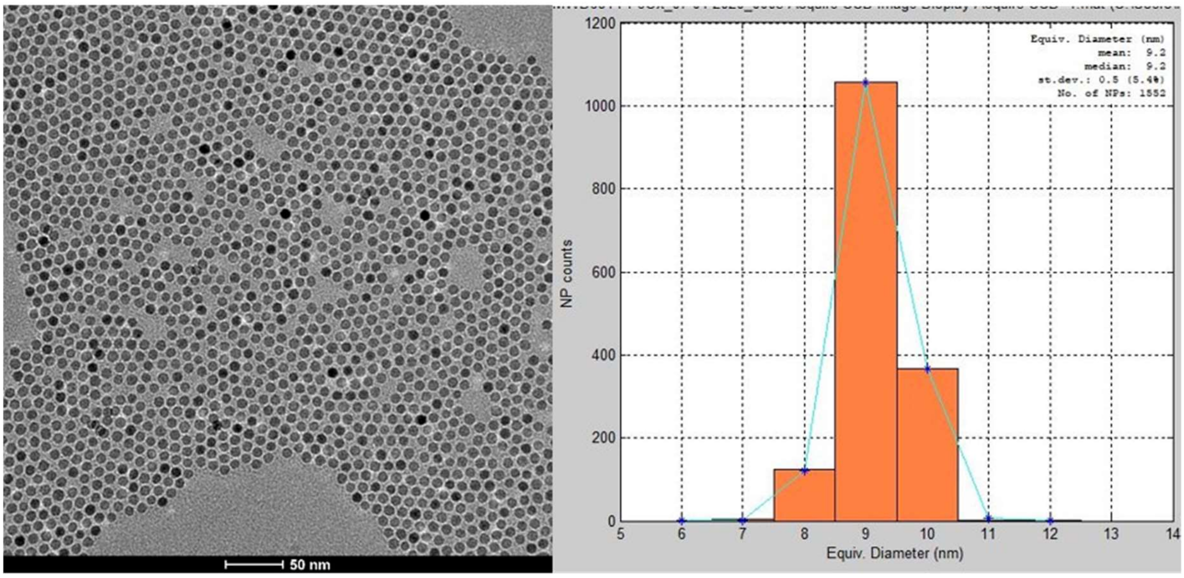

(b)

Figure S1. Size distribution of iron oxide nanoparticles, measured with TEM and calculated with the freeware Pebbles, (a) 3.7 nm (b) 9.2 nm, both scale bars corresponds to 50 nm.

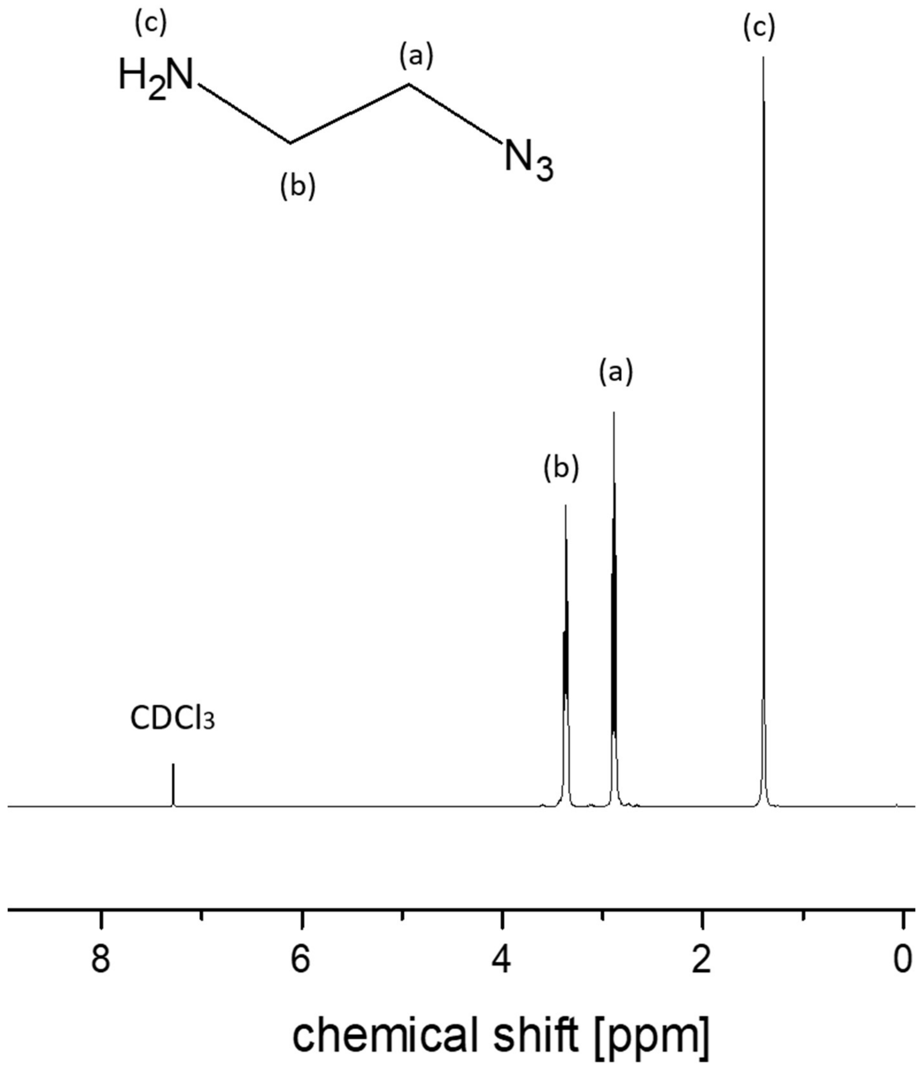

Figure S2. ^{1}H-NMR (300 MHz; $CDCl_3$) of 2-azidoethanamine.

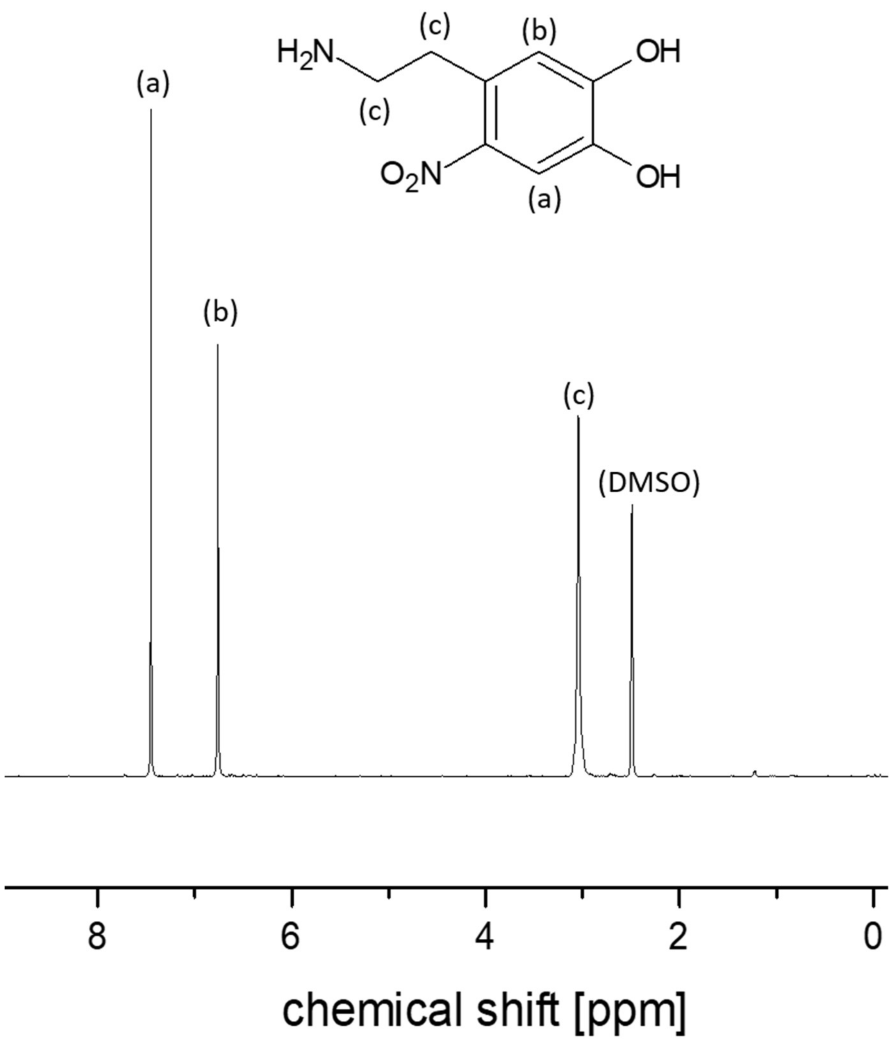

Figure S3. ^{1}H-NMR (300 MHz; DMSO-d$_6$) of 6-nitrodopamine.

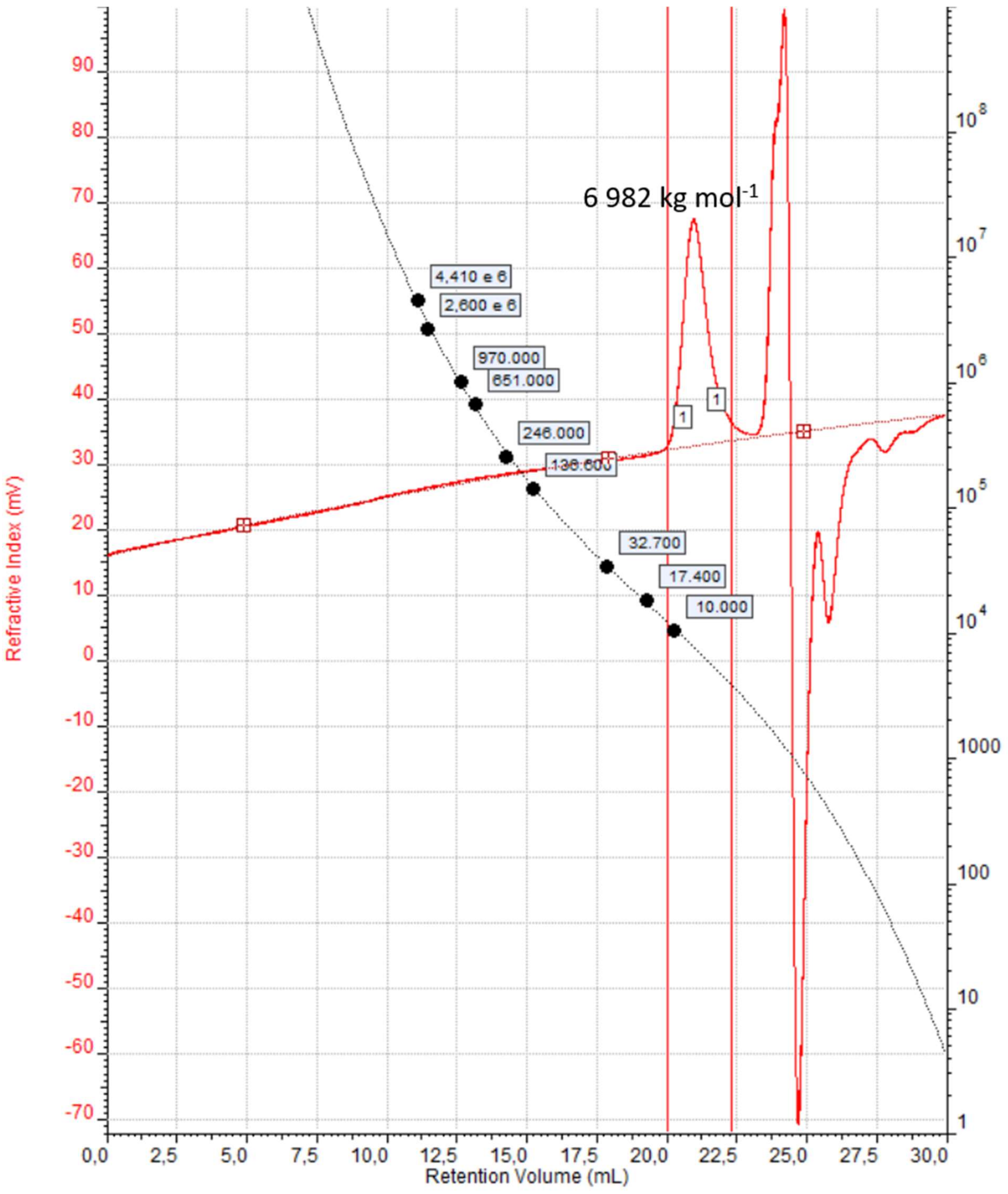
6 982 kg mol-1
4,410 e 6
2,600 e 6
970.000
651.000
246.000
136.600
32.700
17.400
10.000
1
1
Refractive Index (mV)
MW
Retention Volume (mL)

(a)

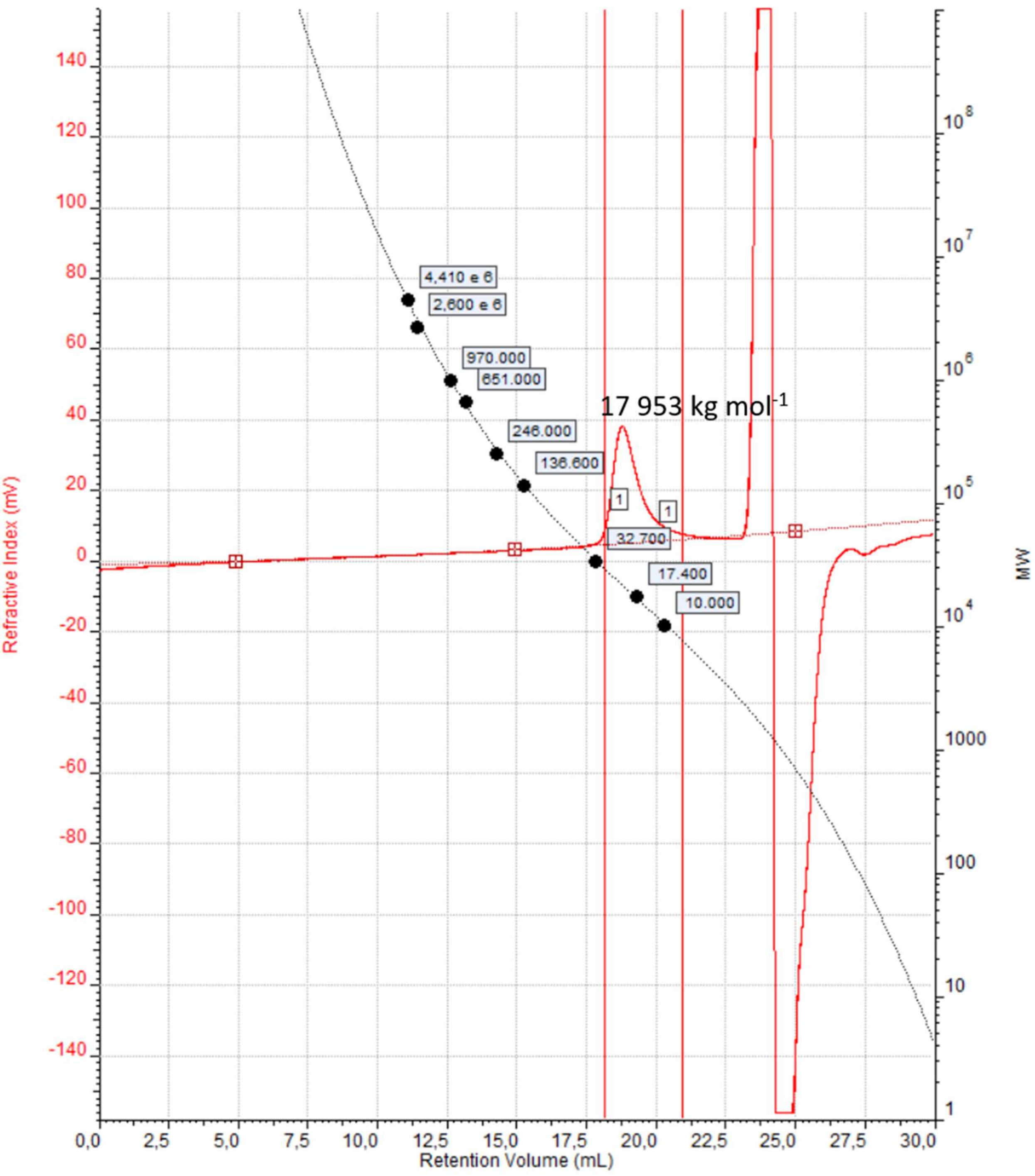

17 953 kg mol-1
4,410 e 6
2,600 e 6
970.000
651.000
246.000
136.600
32.700
17.400
10.000
1
1
Refractive Index (mV)
MW
Retention Volume (mL)

(b)

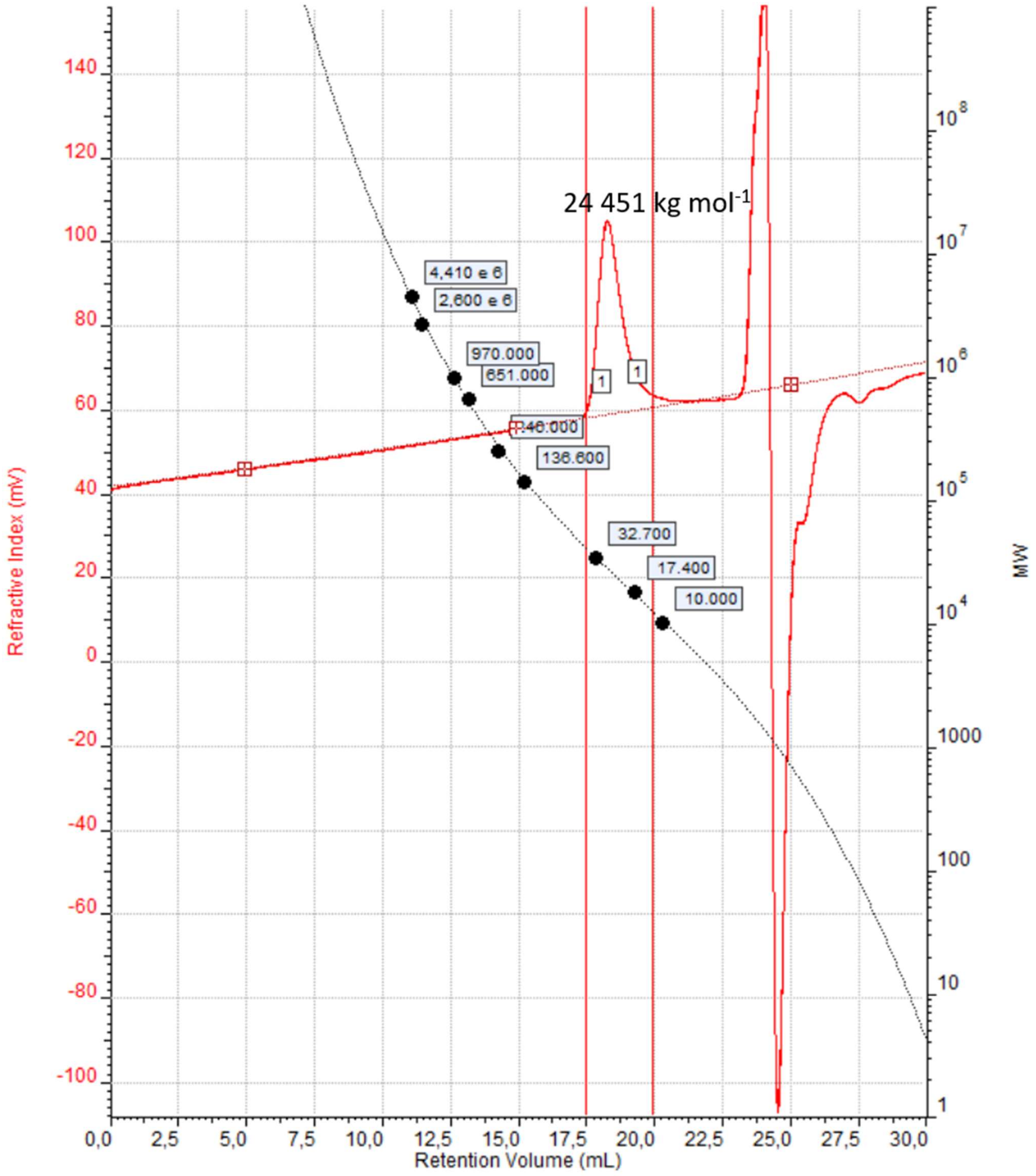

(c)

Figure S4. Gel permeation chromatograms of linear poly(2-isopropyl-2-oxazoline): a) 7 kg mol^{-1}, b) 18 kg mol^{-1}, and c) 24.5 kg mol^{-1}.

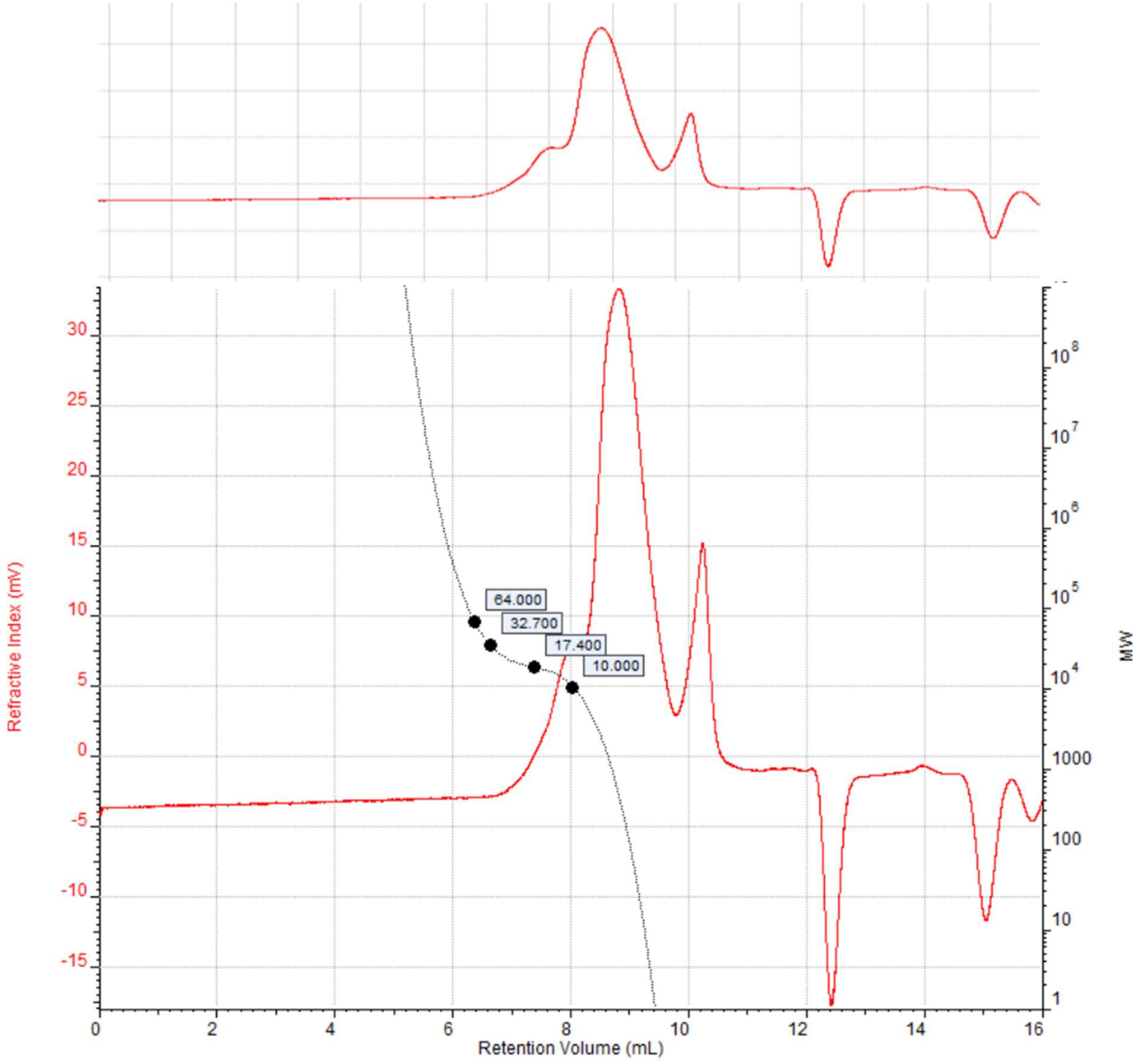

Refractive Index (mV)
30
25
20
15
10
5
0
-5
-10
-15
MW
10⁸
10⁷
10⁶
10⁵
10⁴
1000
100
10
1
64.000
32.700
17.400
10.000
0
2
4
6
8
10
12
14
16
Retention Volume (mL)

(a)

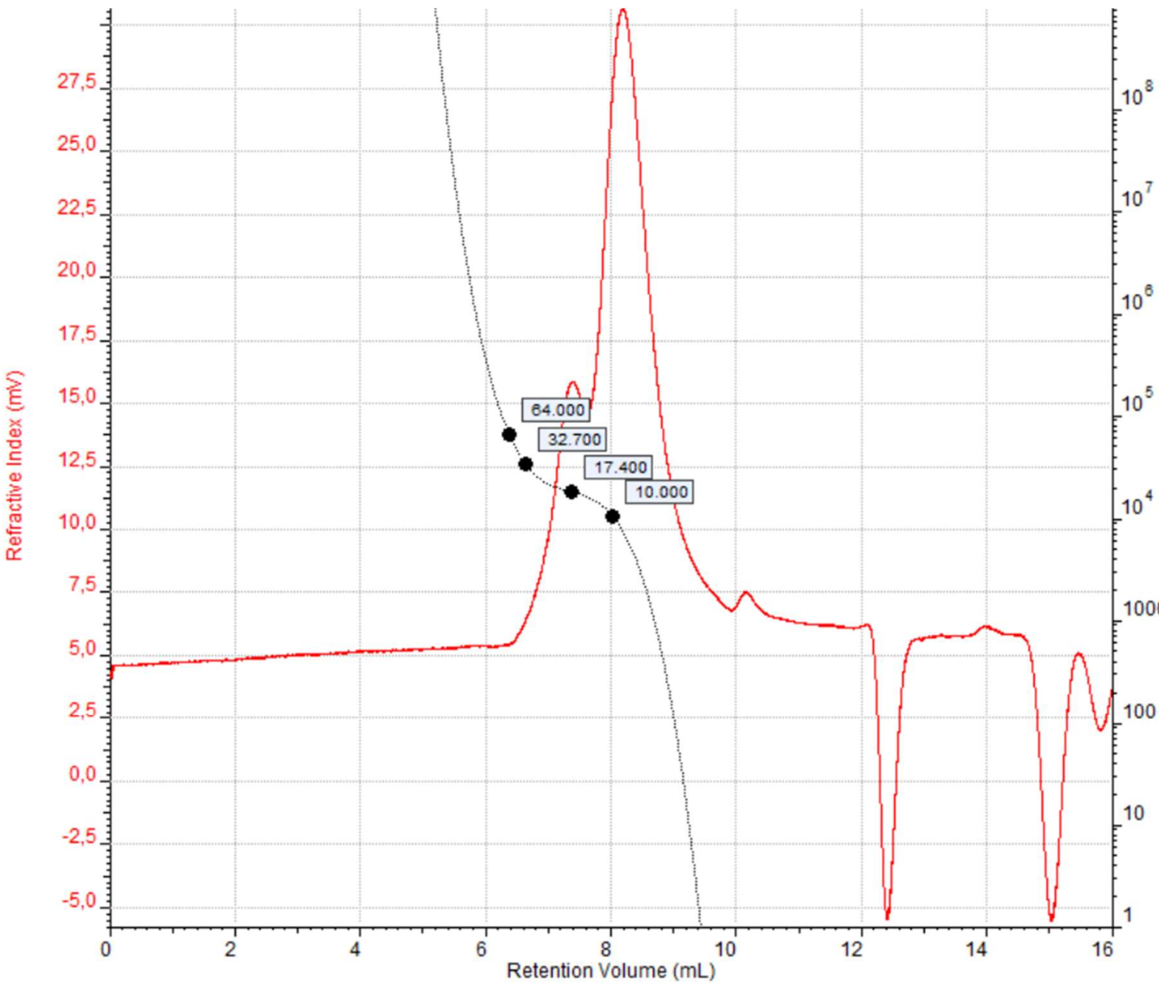
27,5
25,0
22,5
20,0
17,5
15,0
12,5
10,0
7,5
5,0
2,5
0,0
-2,5
-5,0
Refractive Index (mV)
64.000
32.700
17.400
10.000
10^8
10^7
10^6
10^5
10^4
1000
100
10
1
MW
0
2
4
6
8
10
12
14
16
Retention Volume (mL)

(b)

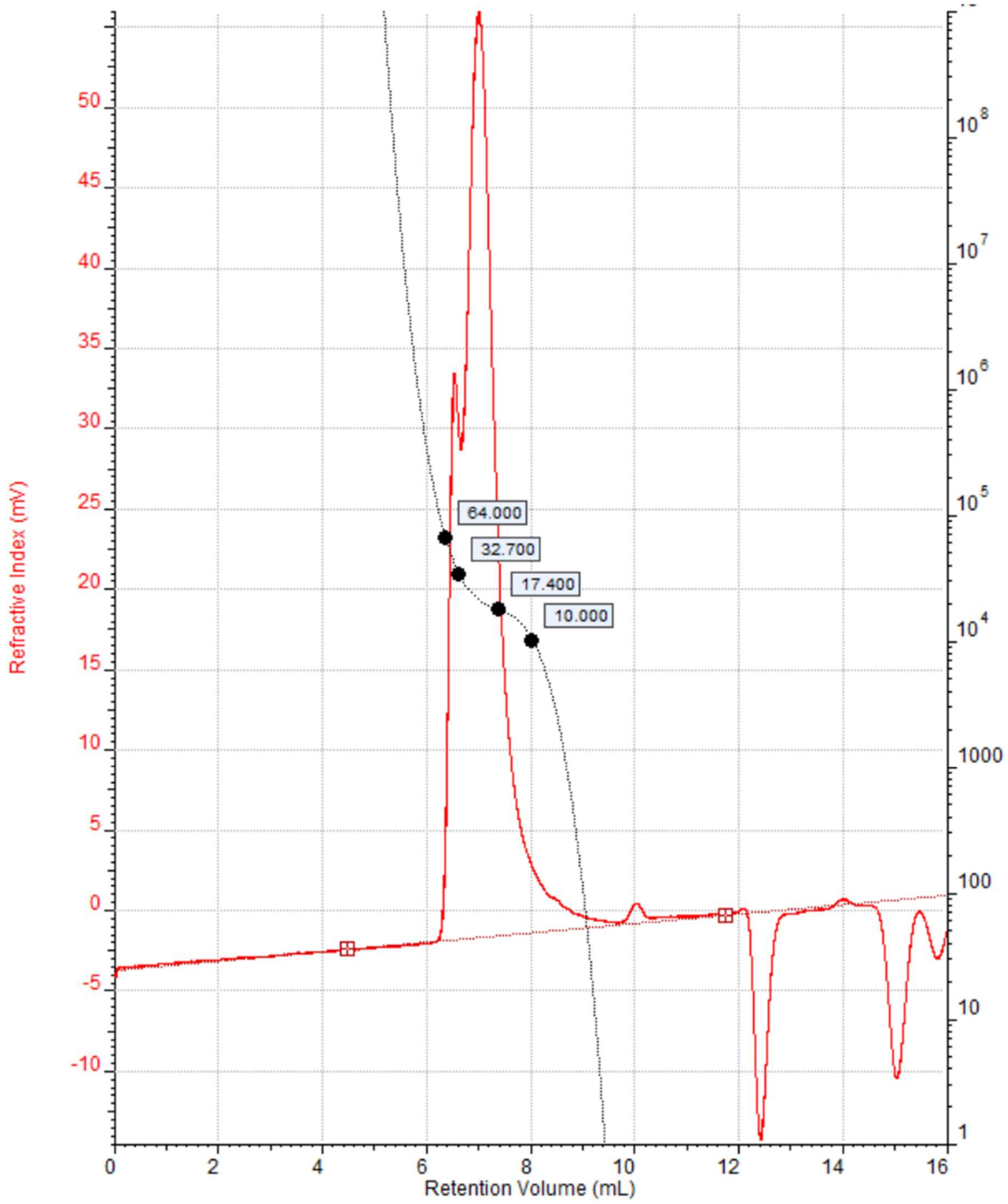

(c)

Figure S5. Gel permeation chromatograms of cyclic poly(2-isopropyl-2-oxazoline): a) 7 kg mol^{-1}, b) 18 kg mol^{-1}, and c) 24.5 kg mol^{-1}. A predominant shift to lower equivalent polystyrene molar mass for cyclic compared to linear polymers support successful cyclization.

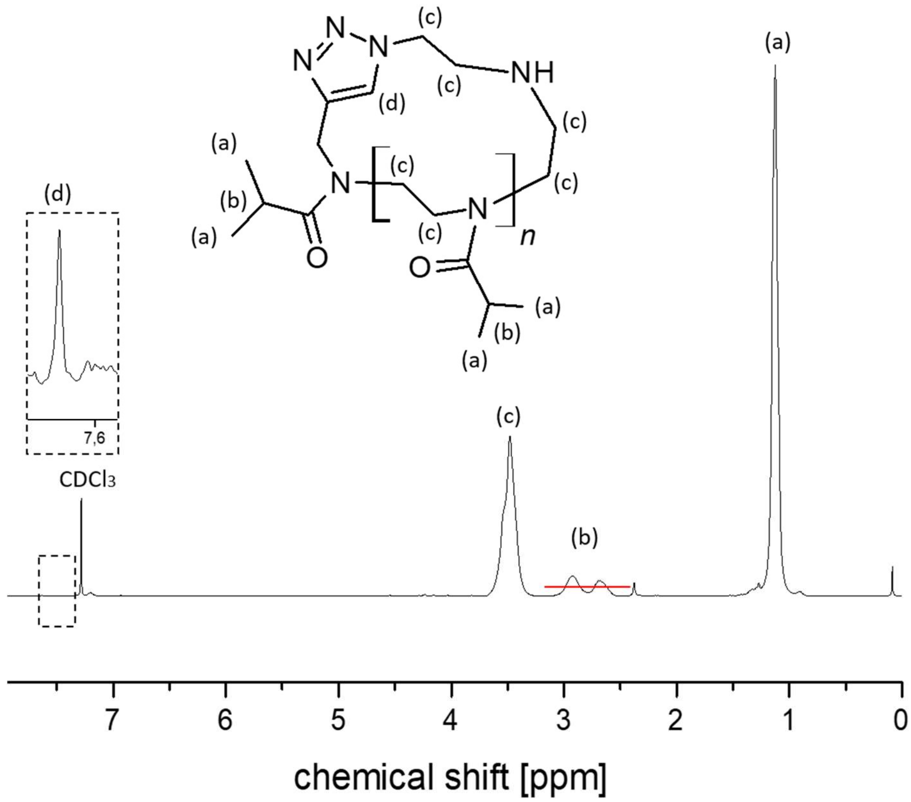
(a)
(b)
(c)
(d)
NH
N
O
n
CDCl3
7,6
chemical shift [ppm]
7
6
5
4
3
2
1
0
(a)

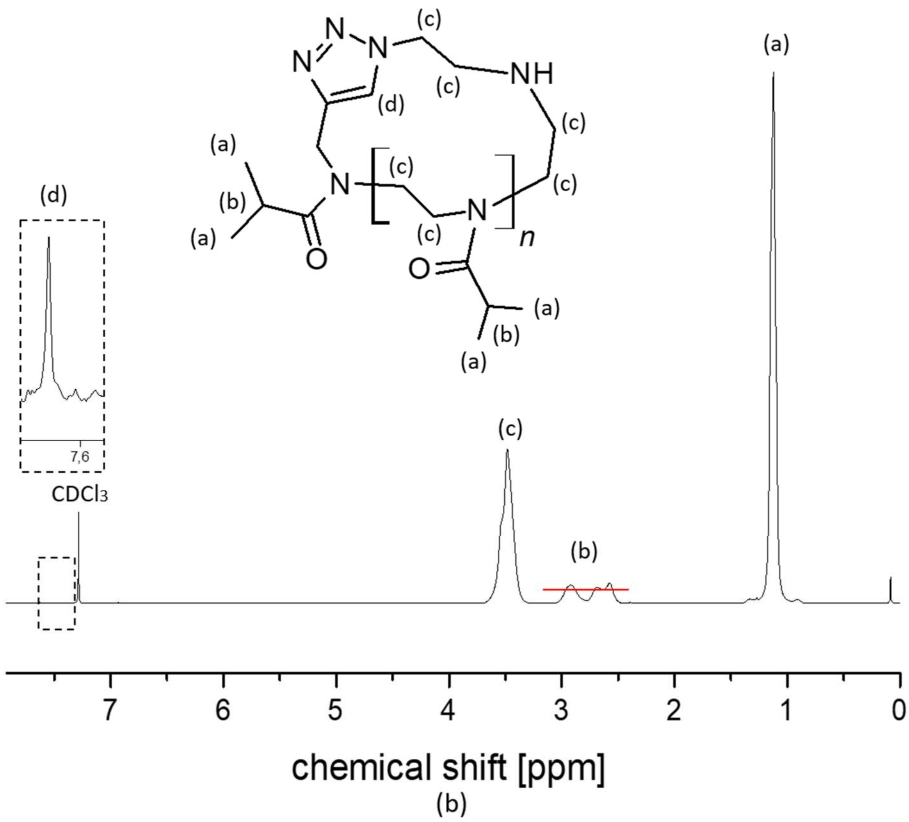
(a)
(b)
(c)
(d)
NH
N
O
n
CDCl3
7,6
chemical shift [ppm]
7
6
5
4
3
2
1
0
(b)

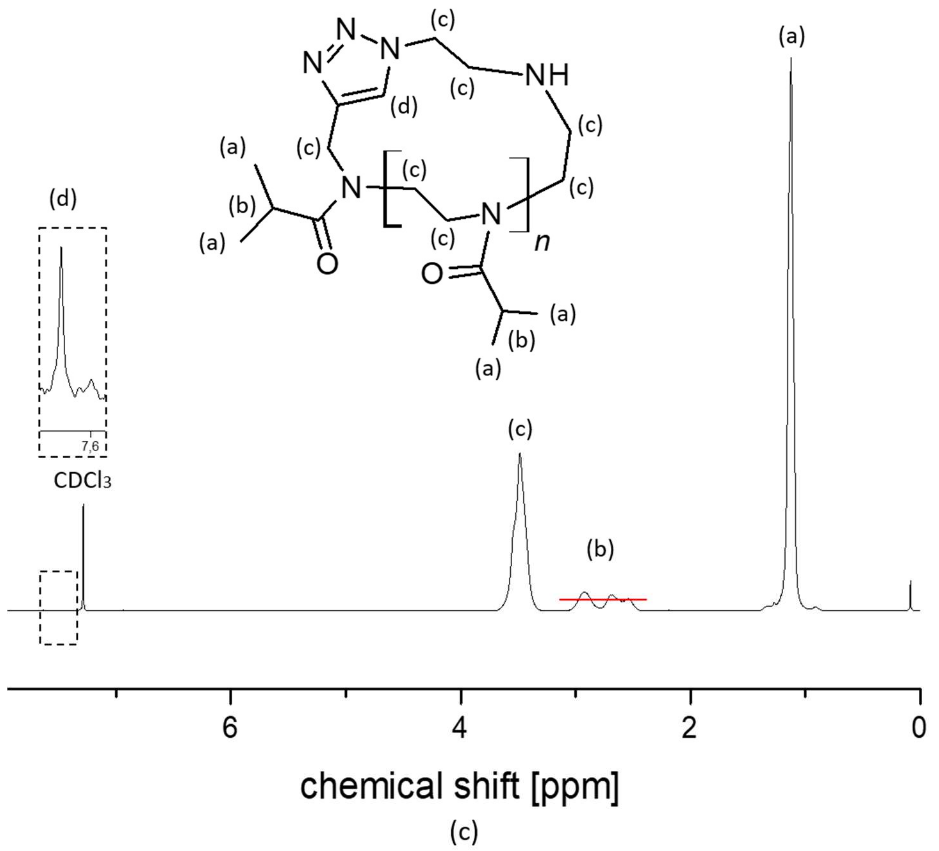

Figure S6. ^{1}H-NMR (300 MHz; $CDCl_3$) of cyclic poly(2-isopropyl-2-oxazoline): a) 7 kg mol^{-1}; b) 18 kg mol^{-1}; c) 24.5 kg mol^{-1}.The symmetry with a slight shoulder at higher ppm of (c) supports the successful cyclization.

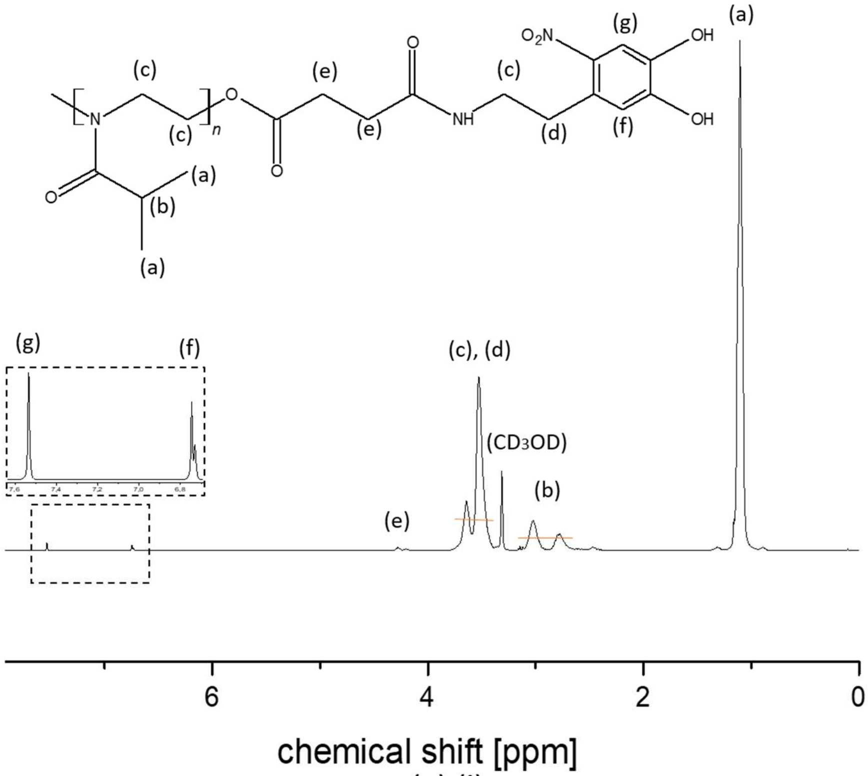
(a)
(g)
O_2N
OH
OH
(c)
(e)
(c)
(e)
NH
(c)
(d)
(f)
n
O
O
O
O
(a)
(b)
(a)
(g)
(f)
(c), (d)
(CD3OD)
(b)
(e)
6
4
2
0
chemical shift [ppm]

(a) (i)

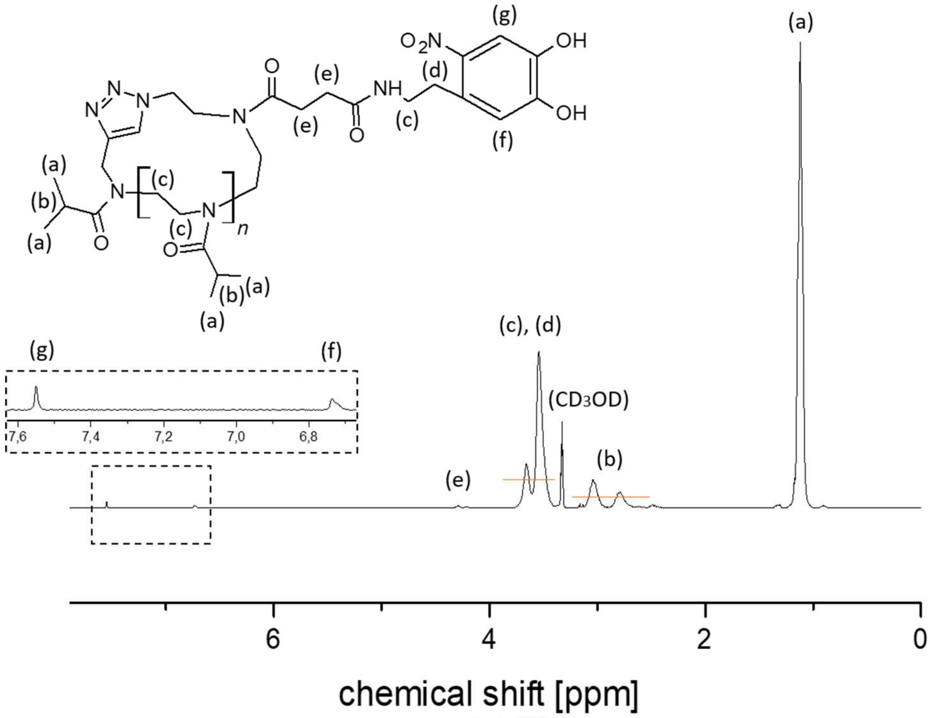
(a)
(g)
O_2N
OH
OH
(e)
NH
(d)
(e)
(c)
(f)
(c)
(c)
n
(a)
(b)
(a)
(b)
(a)
(a)
(g)
(f)
(c), (d)
(CD3OD)
(b)
(e)
6
4
2
0
chemical shift [ppm]

(a) (ii)

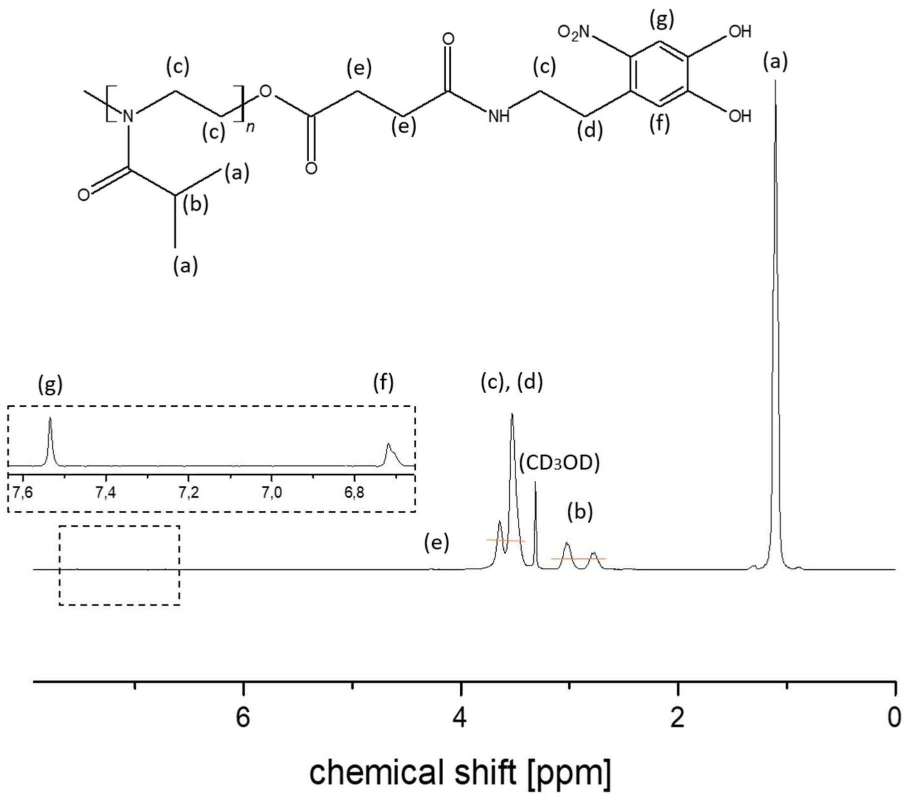

(a)
(b)
(c)
(d)
(e)
(f)
(g)
(c), (d)
(CD3OD)
7,6
7,4
7,2
7,0
6,8
6
4
2
0
chemical shift [ppm]

(b) (i)

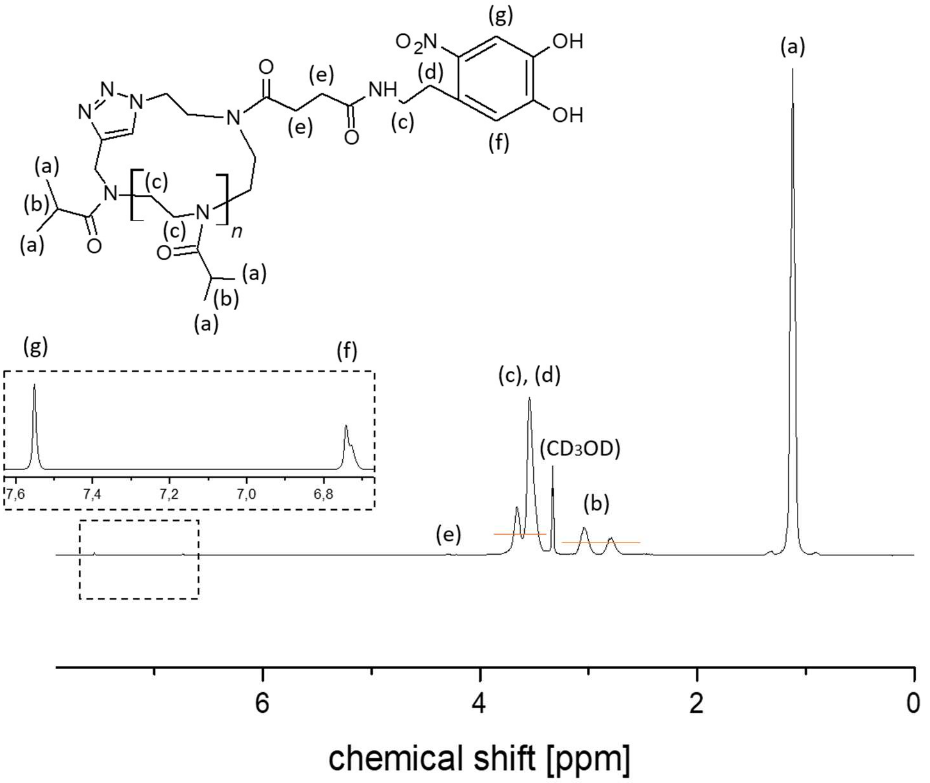

(a)
(b)
(c)
(d)
(e)
(f)
(g)
(c), (d)
(CD3OD)
7,6
7,4
7,2
7,0
6,8
6
4
2
0
chemical shift [ppm]

(b) (ii)

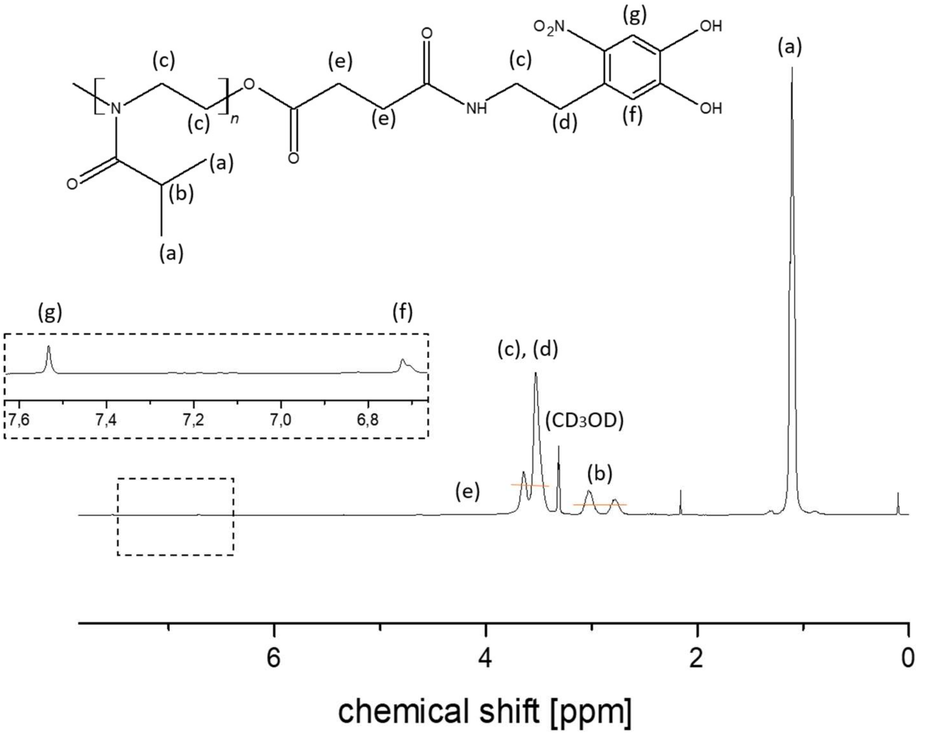

(c) (i)

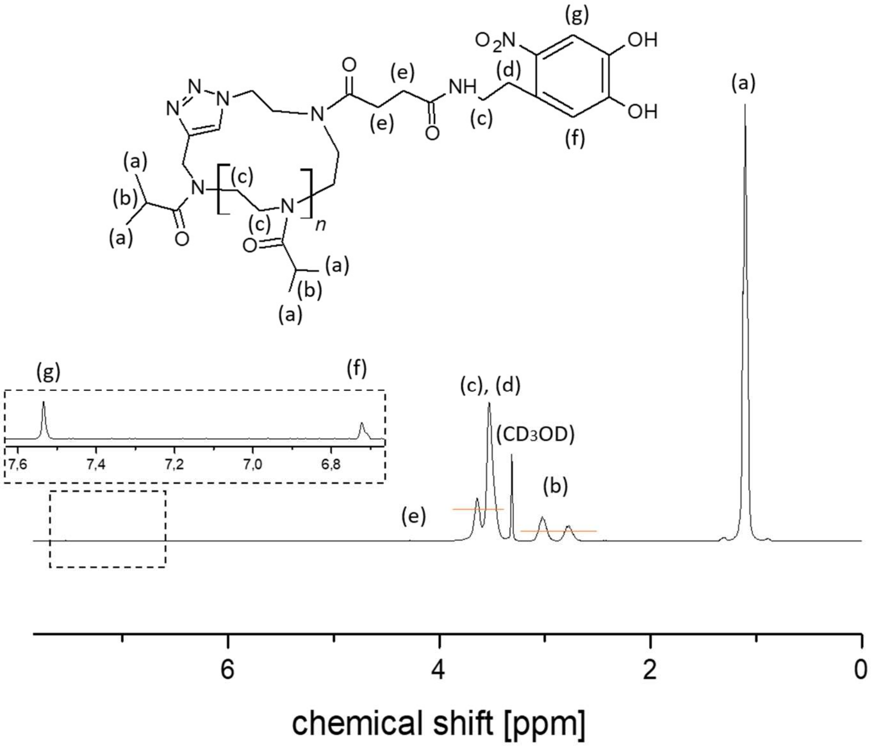

(c) (ii)

Figure S7. ^{1}H-NMR (300 MHz; CD3OD) of 6-nitrodopamine modified linear and cyclic poly(2-isopropyl-2-oxazoline): a) 7 kg mol^{-1} (i) linear, (ii) cyclic; b) 18 kg mol^{-1} (i) linear, (ii) cyclic; c) 24.5 kg mol^{-1} (i) linear, (ii) cyclic.

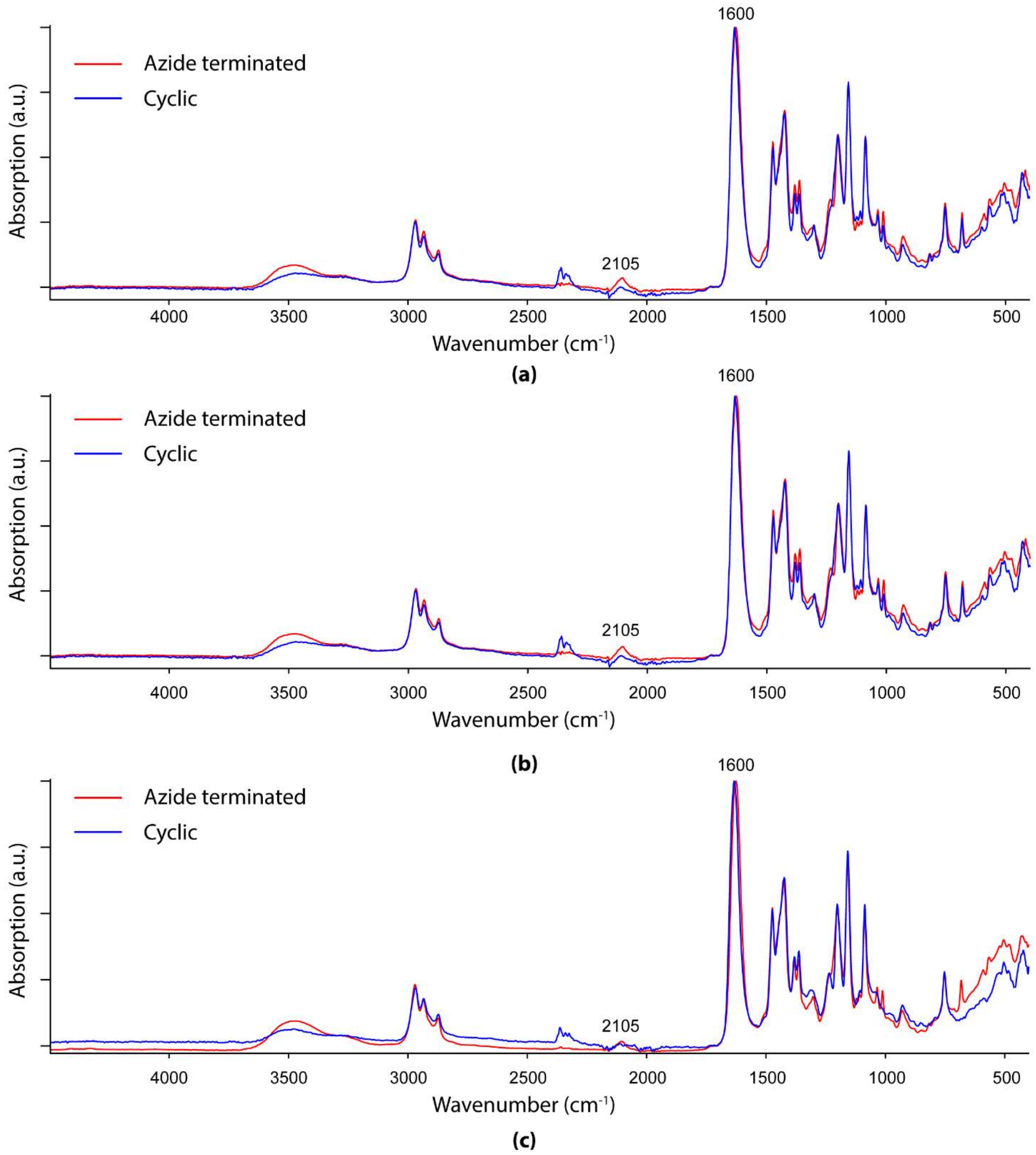

Figure S8. ATR-FTIR spectra of linear (azide-terminated) poly(2-isopropyl-2-oxazoline) before cyclization and cyclic poly(2-isopropyl-2-oxazoline): (a) 7 kg mol^{-1}, (b) 18 kg mol^{-1}, (c) 24.5 kg mol^{-1} (i) linear, (ii) cyclic. Few modes are distinct between the polymers due to the high similarity of their chemical groups. Mainly, the N=N anti/asymmetric peak (including the azide) at 2105 and the N-N stretch peak at 1050 are affected by the cyclization forming the triazole ring.

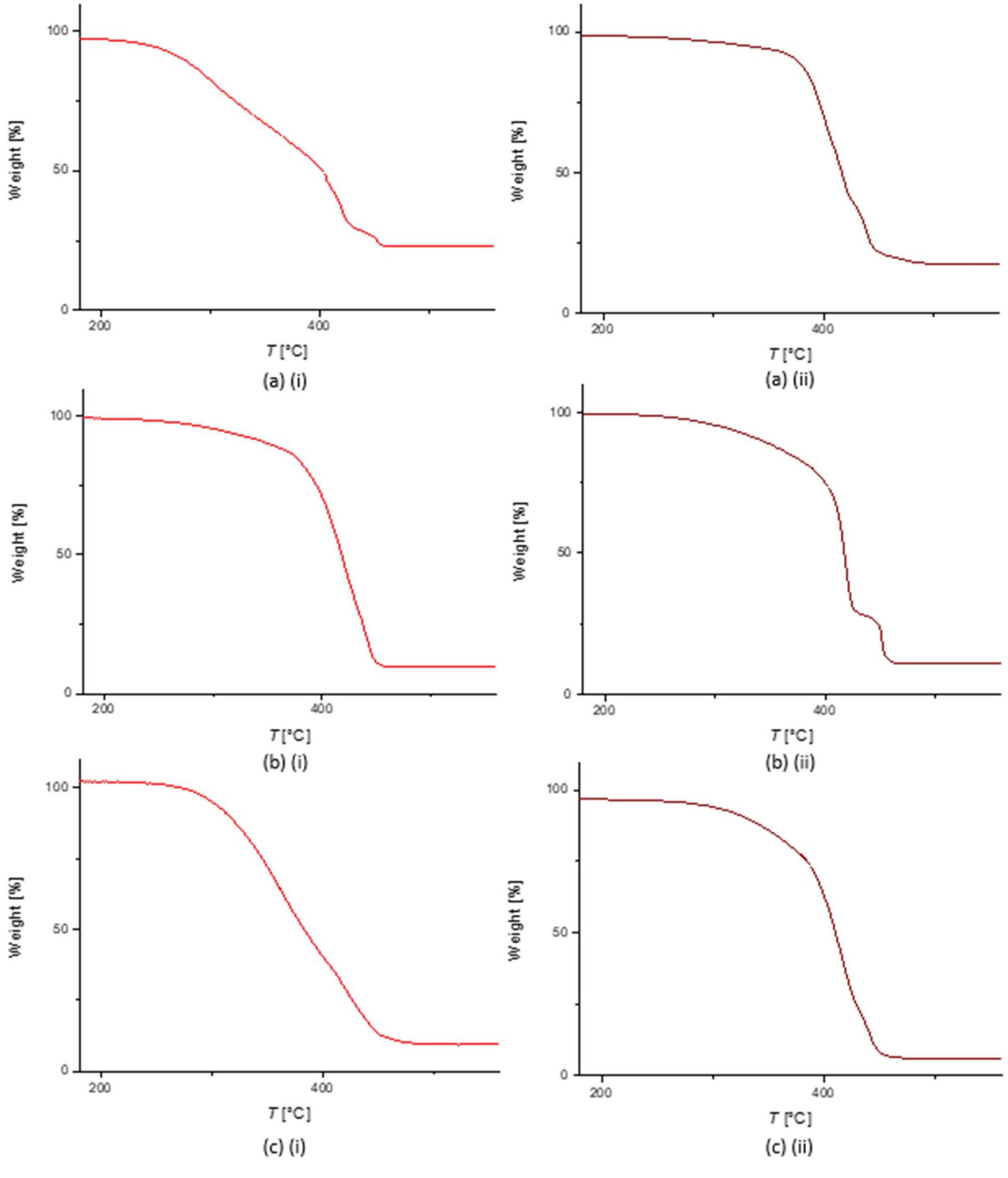

Figure S9. TGA of 3.7-nm core size PiPrOx-grafted core-shell nanoparticles with (a) 7 kg mol^{-1} (b) 18 kg mol^{-1} (c) 24.5 kg mol^{-1} (i) linear (ii) cyclic PiPrOx.

#

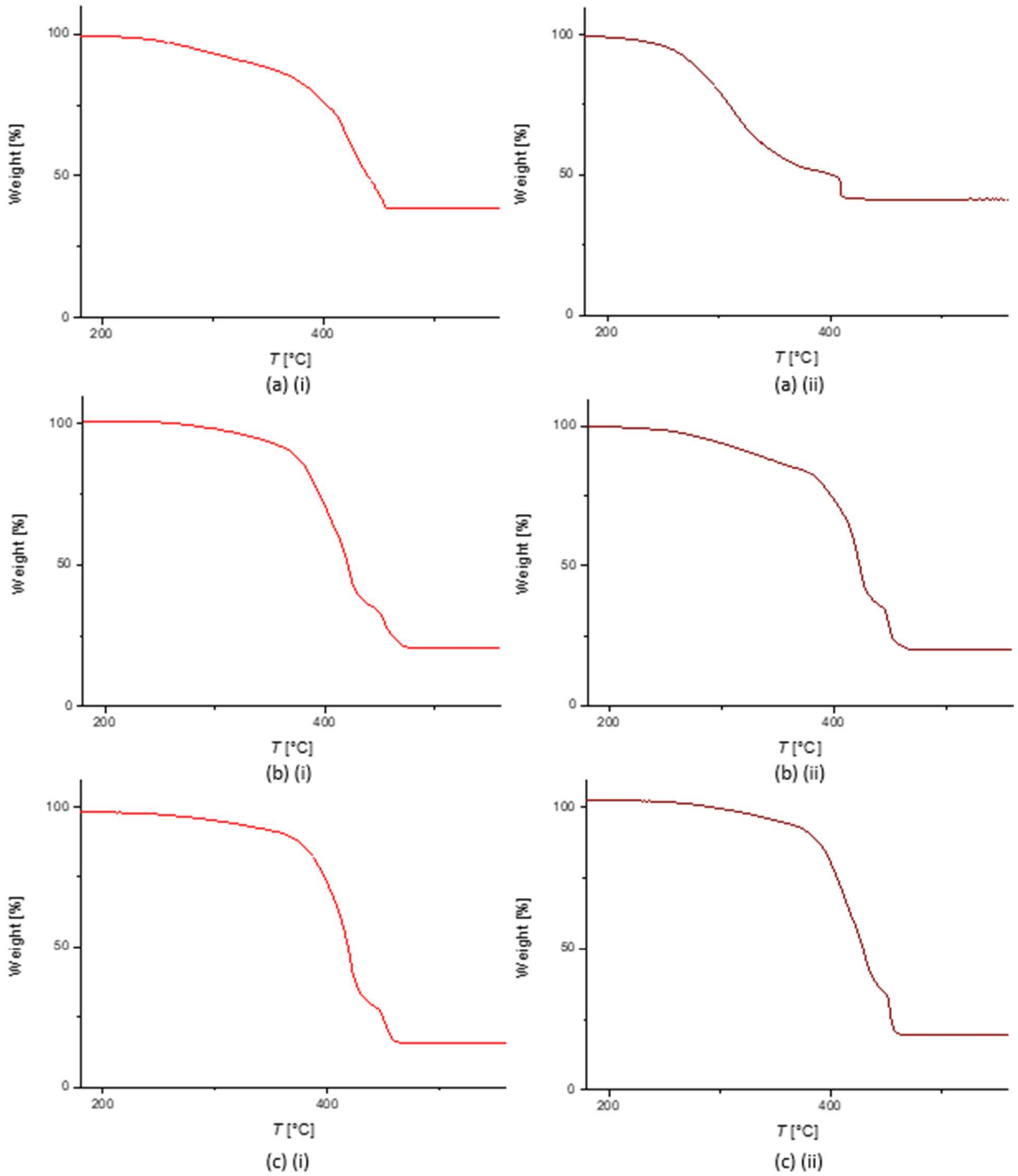

Figure S10. TGA of 9.2-nm core size PiPrOx-grafted core-shell nanoparticles with (a) 7 kg mol^{-1} (b) 18 kg mol^{-1} (c) 24.5 kg mol^{-1} (i) linear (ii) cyclic PiPrOx.

Table S1. TGA results for poly(2-isopropyl-2-oxazoline)-grafted superparamagnetic iron oxide nanoparticles.

Sample	TGA		
	Weight Loss [mg]	Residue [mg]	σ [molecules nm^{-2}]
C4MW7-linear	0.81	0.25	0.9
C4MW7-cyclic	1.08	0.26	1.1
C4MW18-linear	1.69	0.18	1.0
C4MW18-cyclic	0.97	0.12	0.9
C4MW25-linear	0.87	0.09	0.8
C4MW25-cyclic	2.44	0.16	1.2
C9MW7-linear	0.95	0.61	1.1
C9MW7-cyclic	0.71	0.51	1.0
C9MW18-linear	0.84	0.22	1.0
C9MW18-cyclic	1.22	0.32	1.0
C9MW25-linear	2.49	0.48	1.0
C9MW25-cyclic	0.62	0.15	0.8

Table S2. Comparison of the hydrodynamic diameter of the PiPrOx-grafted nanoparticles before and after the temperature cycle and the CFT of each particle dispersion extracted from the data in Figure 3. The CFT is chosen as the onset of the transition in the DLS count rate curve.

Sample	$D_{H,before}$ [nm]	$D_{H,after}$ [nm]	CFT [°C]
Free Polymer MW7-linear	2.7 ± 0.3	656 ± 453	52
Free Polymer MW7-cyclic	3.6 ± 9.4	2.7 ± 0.4	49
Free Polymer MW18-linear	4.0 ± 0.3	4.1 ± 0.2	41
Free Polymer MW18-cyclic	3.9 ± 0.4	4.0 ± 0.4	40
Free Polymer MW25-linear	19.4 ± 4.3	21.8 ± 4.8	39
Free Polymer MW25-cyclic	5.3 ± 0.6	5.4 ± 0.4	37
C4MW7-linear	12.5 ± 2.8	10.1 ± 2.0	42
C4MW7-cyclic	69.0 ± 8.2	75.4 ± 12.9	43
C4MW18-linear	17.1 ± 0.9	17.2 ± 0.8	35
C4MW18-cyclic	122.3 ± 9.6	134.1 ± 5.3	36
C4MW25-linear	17.7 ± 3.0	19.6 ± 2.7	35
C4MW25-cyclic	115.3 ± 8.1	127.8 ± 5.7	37
C9MW7-linear	20.3 ± 1.6	21.1 ± 2.8	33
C9MW7-cyclic	205 ± 120	179 ± 14	31
C9MW18-linear	18.7 ± 4.3	29.4 ± 1.2	36
C9MW18-cyclic	97.0 ± 10.6	111.2 ± 5.4	39
C9MW25-linear	20.6 ± 2.4	25.3 ± 2.5	36
C9MW25-cyclic	87.9 ± 3.9	101.1 ± 2.4	38
